# In situ protein corona–camouflaged supramolecular assemblies remodel thrombotic microenvironment for improved arterial homeostasis

**DOI:** 10.1126/sciadv.adu6676

**Published:** 2025-05-02

**Authors:** Dan Chen, Yifan Chen, Jianwen Liu, Xinyue Liu, Peiwen Liu, Jiabing Zhan, Zhiting Chen, Yong Gan, Mingdong Huang, Zhaoyang Chen

**Affiliations:** ^1^ Department of Cardiology, Heart Center of Fujian Province, Fujian Medical University Union Hospital, Fuzhou, Fujian 350001, China.; ^2^ Department of Cardiology, Xiamen Cardiovascular Hospital, Xiamen University, Xiamen, Fujian 361004, China.; ^3^ College of Chemistry, Fuzhou University, Fuzhou, Fujian 350116, China.; ^4^ Department of Neurology, Fujian Medical University Union Hospital, Fuzhou, Fujian 350001, China.; ^5^ Shanghai Institute of Materia Medica, Chinese Academy of Sciences, Shanghai 201203, China.; ^6^ School of Pharmacy, University of Chinese Academy of Sciences, Beijing 100049, China.

## Abstract

Arterial thrombosis is commonly accompanied by poor recanalization and high recurrence, typically caused by a fibrinolysis-resistant microenvironment. We identify elevated levels of plasminogen activator inhibitor–1 (PAI-1) and, notably, its strong correlation with inflammation in arterial thrombosis. To address this, small molecular inhibitors of PAI-1 and inflammation are used as bioregulators to restore vascular homeostasis. We design a carrier-free supramolecular system based on the bioregulators-tuned self-assembly of a near-infrared thrombus probe, which preferentially forms protein corona in situ to enhance plasma stability. Under acidic conditions and increased shear stress, the supramolecular assemblies disintegrate, enabling site-specific cargo release. In vivo, the probe accumulates 22.8-fold more in the thrombotic than contralateral artery. Functionally, this nanomedicine improves outcomes in mice with carotid artery thrombosis and chronic cerebral ischemia. Mechanistically, it down-regulates NF-κB signaling, inhibits NETosis and glycolysis, and up-regulates cGMP-mediated signaling, thereby alleviating inflammation and promoting fibrinolysis. This study offers an innovative codelivery strategy using supramolecular assemblies to advance therapies for arterial thrombosis.

## Teaser

Supramolecular assemblies codeliver bioregulators to target inflammation and fibrinolysis in arterial thrombosis.

## INTRODUCTION

Arterial thrombotic events contribute to several cardiovascular diseases, including ischemic stroke and myocardial infarction, resulting in substantial disability and premature death (*1*–*3*). Plasminogen activator (PA)–based systemic thrombolytic therapy is one of the mainstream approaches for thrombus removal and vessel recanalization. However, it faces several challenges, including severe hemorrhagic transformation, ineffective recanalization, and a high risk of reocclusion (*4*, *5*). Clinical evidences indicate that the recanalization rate in patients with stroke treated with recombinant tissue-type PA (rtPA) is around 34%, which further drops to 6% in patients with platelet-rich thrombi (*6*, *7*). Alarmingly, the rate of secondary reocclusion reaches 14 to 34% due to increased platelet aggregation following thrombolysis (*6*). Moreover, arterial thrombolysis outside the context of ischemic stroke and ST-elevation myocardial infarction necessitates patient risk stratification and is typically limited to catheter-based interventions (*8*, *9*). These challenges underscore the need for novel therapeutic strategies targeting the unique features of arterial thrombotic microenvironment.

Vascular homeostasis is a critical determinant of thrombotic disease outcomes. Arterial thrombosis is closely linked to endothelial damage and impaired fibrinolysis (*10*–*12*). Moreover, inflammation, a well-recognized driver of thrombosis, has been revealed to exacerbate endothelial dysfunction and coagulopathy (*13*, *14*). Recent clinical trials have demonstrated the therapeutic benefits of anti-inflammatory interventions, highlighting the critical role of inflammation in arterial thrombotic microenvironment (*15*, *16*). These findings suggest that simultaneously targeting the fibrinolytic pathway and inflammation, with improved endothelial function, represents a promising strategy in treating arterial thrombotic conditions.

Natural small molecules (NSMs) hold great potential for regulating disease microenvironment (*17*, *18*). However, their clinical applications are hindered by low solubility, poor stability, and limited bioavailability. Although various delivery vectors have been developed in recent years to enhance the in vivo delivery of NSMs, these systems often face limitations including complex preparation processes, restricted drug loading capacity, and potential safety hazard, which collectively constrain their clinical utility (*19*). Recently, carrier-free supramolecular systems, assembled from NSMs through multiple noncovalent interactions, have garnered substantial attention for their ability to enhance drug loading capacity, bioavailability, and therapeutic precision while minimizing off-target effects (*20*, *21*). Embelin, a traditional herbal compound, has been identified in our previous studies as a micromolar inhibitor of plasminogen activator inhibitor–1 (PAI-1) [median inhibitory concentration (IC_50_) = 1.6 μM], the primary cause of fibrinolytic resistance in platelet-rich arterial thrombi (*22*, *23*). Notably, embelin has shown efficacy in improving coagulopathy in an lipopolysaccharide (LPS)–induced murine sepsis model (*24*). Besides, isoquercetin, a flavonoid with anti-inflammatory and antithrombotic effects, has demonstrated clinical success in reducing thrombotic risk in patients with advanced cancer (NCT02195232) (*25*). Based on these basic and clinical studies, we hypothesize that codelivery of embelin and isoquercetin within a self-assembled supramolecular system could simultaneously target fibrinolytic pathway and excessive inflammation. While supramolecular systems involve single-component, two-component, or multicomponent approaches, the field is largely still driven by random findings and serendipity (*21*, *26*). There is a need for a goal-oriented design with a streamlined preparation process. Furthermore, these carrier-free systems, stabilized by weak noncovalent interactions, often suffer from poor stability in complex in vivo environments. Given that supramolecular self-assembly is an emerging technology, only limited research has addressed these challenges (*27*).

**Fig. 1.**
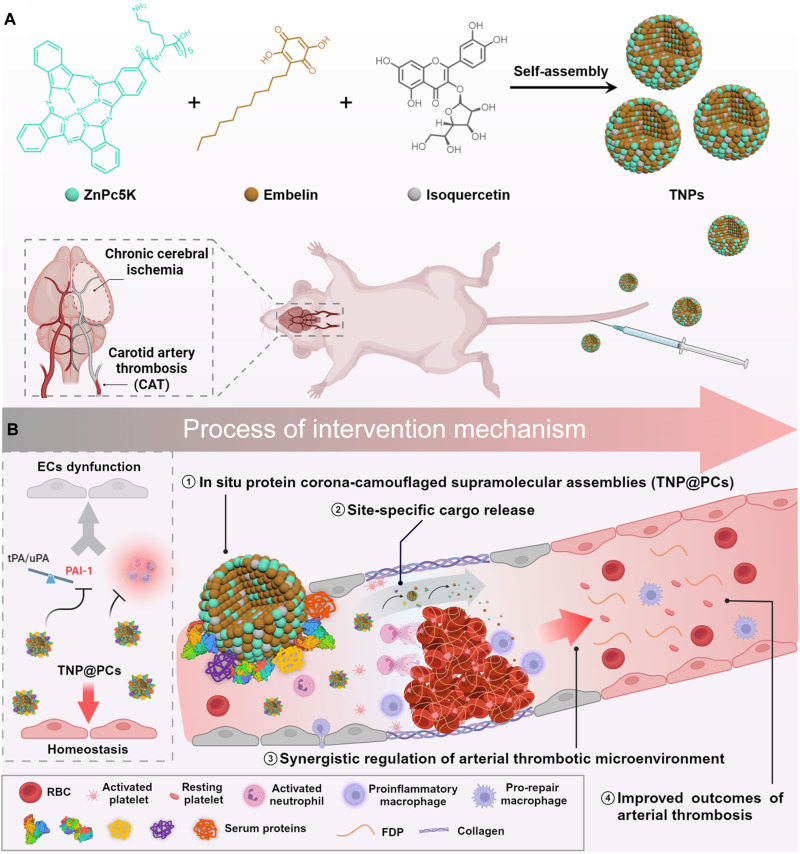
Schematic diagram illustrating the design and proposed intervention mechanism of TNP@PCs for treating arterial thrombosis. (**A**) Supramolecular assembly of ZnPc5K, embelin, and isoquercetin into TNPs for the treatment of carotid artery thrombosis (CAT) and subsequent chronic cerebral ischemia. (**B**) Proposed intervention mechanism of TNP@PCs in treating arterial thrombosis. Created in BioRender (Chen, D., 2025; https://BioRender.com/m06r466).

Here, we demonstrate that PAI-1 in its active conformation is closely correlated with systemic inflammation in the context of arterial thrombosis. A considerable amount of PAI-1 protein localizes in the inflamed areas populated by abundant myeloperoxidase (MPO)–positive cells. We then introduce a near-infrared fluorescent probe to enable the spontaneous formation of supramolecular assemblies with embelin and isoquercetin [ternary nanoparticles (TNPs)]. The probe has an amphiphilic structure with a hydrophobic phthalocyanine and a hydrophilic pentalysine moiety, exhibiting strong self-assembly capacity (*28*–*30*). In addition, the probe demonstrates biocompatibility and is capable of binding to thrombi without potentially affecting the process of thrombus formation and dissolution (*31*, *32*). TNPs with positively charged surface promptly attracts plasma proteins and forms a protein corona (PC) onto the nanostructure in situ (designated TNP@PCs), thereby enhancing the stability in systemic circulation. Furthermore, the NSM-tuned supramolecular assembly traps the probe in a dark state, enabling it to function as an indicator of the disintegration of the supramolecular system. In vivo, TNP@PCs are disrupted under acidic conditions and elevated shear stress induced by arterial thrombosis, resulting in the desorption of the coronal proteins, the breakdown of nano-assemblies, and the visual identification of nearby thrombus. The bioregulators released during this process remodel the prothrombotic microenvironment by inhibiting nuclear factor κB (NF-κB) signaling, NETosis, and glycolysis while promoting cyclic guanosine 3′,5′-monophosphate (cGMP)–mediated signaling, leading to a synergistic enhancement of therapeutic outcomes in both in situ carotid artery thrombosis and distal cerebral ischemia. Moreover, short-term and long-term safety assessments show that TNP@PCs exhibit favorable biosafety without bleeding complications. Furthermore, this in situ PC-camouflaged nanomedicine, featuring site-specific cargo release, provides a unique approach for designing carrier-free supramolecular systems that combine plasma stability with dual responsiveness for interventions in arterial thrombosis (Fig. 1).

## RESULTS

### Positive correlation between PAI-1 expression and inflammation in arterial thrombosis

**Fig. 2.**
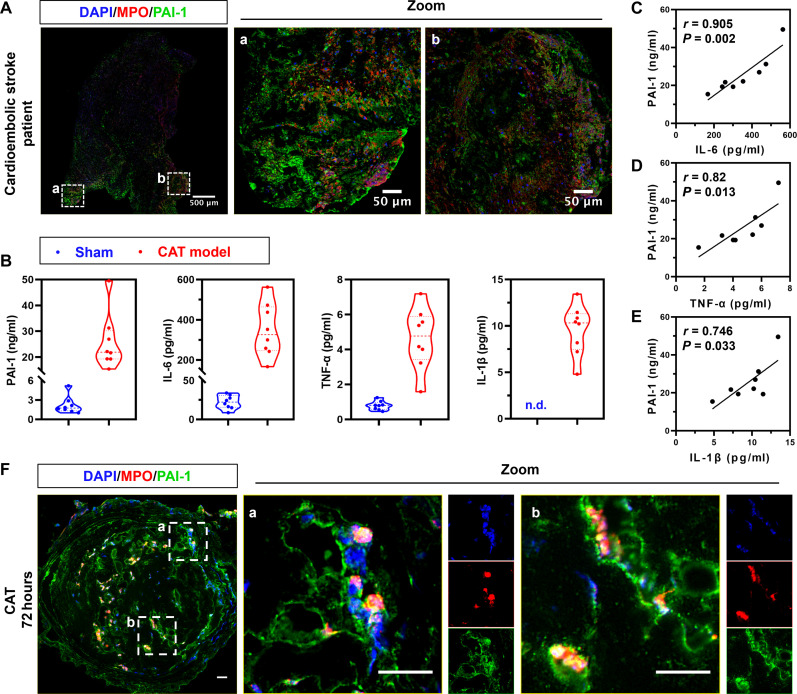
Positive correlation between PAI-1 expression and inflammation in arterial thrombotic microenvironment. (**A**) Representative images of immunostained FFPE sections of clots from patients with cardiogenic stroke (*n* = 2 participants). (**B**) Plasma levels of active PAI-1, IL-6, TNF-α, and IL-1β in sham-operated and CAT mice at 24 hours post-surgery (n.d.: not detectable, *n* = 8 mice). (**C** to **E**) Correlation between plasma levels of active PAI-1 and inflammatory cytokines in CAT mice at 24 hours post-surgery, along with Pearson’s correlation coefficient and the respective *P* values. (**F**) Representative images of immunostained carotid arteries of CAT mice at 72 hours post-surgery. Scale bars, 20 μm.

PAI-1 is widely recognized as a pivotal contributor to thrombolytic resistance, especially in platelet-rich arterial thrombus. Recent studies highlight the substantial involvement of innate immune cells in thrombus formation (*16*, *33*). However, the potential correlation between PAI-1, especially in its active form, and inflammation in the pathogenesis and prognosis of arterial thrombotic disorders remains incompletely elucidated. We analyzed formalin-fixed paraffin-embedded (FFPE) sections of clots retrieved from two patients with cardiogenic stroke and found prominent PAI-1 staining in areas with numerous MPO-positive cells (Fig. 2A and figs. S1 and S2). In mice with arterial thrombi induced by carotid artery injury, plasma levels of active PAI-1, interleukin-6 (IL-6), tumor necrosis factor–α (TNF-α), and IL-1β were significantly higher than those in sham-operated mice at 24 hours post-injury (Fig. 2B). Further Pearson’s correlation analysis showed a significantly positive correlation between the level of active PAI-1 and proinflammatory cytokines, including IL-6, TNF-α, and IL-1β (*r* = 0.905, *P* = 0.002; *r* = 0.82, *P* = 0.013; and *r* = 0.746, *P* = 0.033, respectively) (Fig. 2, C to E). Similar to human arterial thrombi, PAI-1 was readily detectable in MPO-positive myeloid cells in injured carotid arteries at 72 hours (Fig. 2F). Our results showed that PAI-1 protein positively correlated with acute inflammation at the early stages of arterial thrombosis and colocalized with neutrophils in the microenvironment of clotted carotid arteries at 72 hours post-injury. Together, it is likely the collaboration of PAI-1 and the local activated innate immune cells leads to resistance of fibrinolysis of blood clots and poor outcomes of arterial thrombosis.

### Preparation and characterization of supramolecular assemblies (TNPs)

**Fig. 3.**
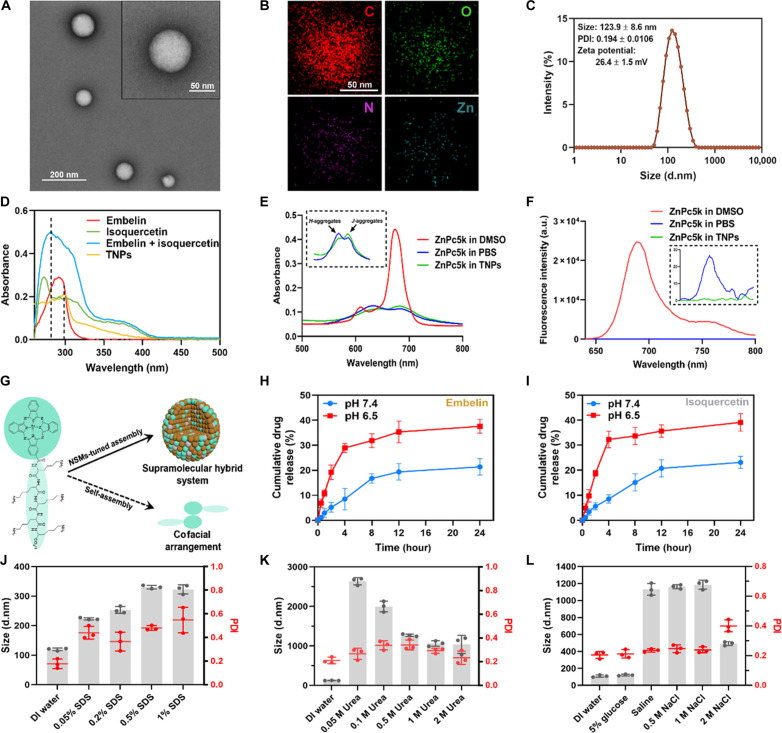
Characterization of TNPs. Representative (**A**) TEM image and (**B**) EDS mappings of TNPs. (**C**) Hydrodynamic diameter of TNPs. (**D**) UV-vis absorption spectra of embelin, isoquercetin, embelin + isoquercetin, and TNPs. (**E**) UV-vis absorption and (**F**) fluorescence spectrum of ZnPc5K in DMSO, PBS, and TNPs, respectively (λ_ex_ = 610 nm). (**G**) Schematic illustration of NSM-tuned supramolecular assembly and ZnPc5K self-assembly in cofacial arrangement. Cumulative release profiles of (**H**) embelin and (**I**) isoquercetin from TNPs at neutral and acidic conditions. Data are means ± SD (*n* = 3 independent samples). Size and polydispersity coefficient (PDI) variations of TNPs incubated with different concentrations of (**J**) SDS, (**K**) urea, and (**L**) NaCl, respectively. Data are means ± SD (*n* = 3 independent samples).

The above finding of key factors contributing to the unsatisfactory therapeutic outcomes in arterial thrombosis prompted the development of a therapeutic nanomedicine capable of efficiently and accurately delivering bioregulators to remodel the prothrombotic microenvironment. To achieve this, we used supramolecular chemistry to coassemble the bioregulators, namely, embelin and isoquercetin, with a thrombus-specific probe, ZnPc5K, which has a strong capacity for self-assembly. In previous studies, we conjugated a pentalysine moiety to β-monocarboxylate–substituted phthalocyanine zinc, obtaining a phthalocyanine derivative with high purity, improved water solubility, and significantly stronger binding affinity to fibrin compared to human serum albumin (HSA) (*28*, *32*). Most supramolecular systems typically consist of two structurally similar molecules in a 1:1 ratio, while the self-assembly of multicomponents has been less explored and lacks precise control over the ratios. Given the presence of multiple functional groups in ZnPc5K and isoquercetin, we investigated the self-assembly process by increasing the molar ratio of embelin to compensate for its limited interaction capacity. Our results indicated that self-assembly into nanostructures occurs when the molar concentration of embelin is three to four times that of ZnPc5K and isoquercetin. Notably, a molar ratio of 1:4:1 for the probe, embelin, and isoquercetin yielded nanostructures with an average particle size of approximately 120 nm, which remained stable for 3 days at room temperature (fig. S3, A and B). Transmission electron microscopy (TEM) showed a spherical morphology of TNPs (Fig. 3A). Elemental mapping revealed abundant carbon, centrally concentrated oxygen, and uniformly distributed nitrogen and zinc in the nanostructure (Fig. 3B and fig. S4). Dynamic light scattering (DLS) results further confirmed the formation of evenly dispersed and positively charged nanoparticles (Fig. 3C). The TNPs fabricated in this study exhibited a high encapsulation efficiency (EE) of 70.0 ± 2.7% and loading capacity (LC) of 61.4 ± 2.3% for embelin, a highly hydrophobic small molecule with long alkyl chains. For isoquercetin, the EE and LC were 11.0 ± 0.9 and 3.8 ± 0.4%, respectively, which may be attributed to the steric hindrance introduced by its glycosyl group. These results highlight a common challenge in carrier-free self-assembled supramolecular system to enhance the encapsulation of glycosylated compounds with steric hindrance while maintaining high loading capacities for highly hydrophobic molecules.

The ultraviolet-visible (UV-vis) spectrum indicated that the maximum absorption peak was red-shifted in the TNPs compared to the embelin and isoquercetin mixture (Fig. 3D). Typically, the fluorescent probe dissolved in dimethyl sulfoxide (DMSO) exists in monomeric form and has a maximum absorption at 678 nm (Q band). In phosphate-buffered saline (PBS), the probe aggregates due to π-π stacking between macrocyclic molecules (cofacial arrangement, *H*-aggregation), leading to a blue shift of the Q band to 630 nm and notably decreased fluorescence (ex_610nm_/em_690nm_). In TNPs, the characteristic absorption peak of the probe was red-shifted compared to its state in PBS, indicating a distinct arrangement of the probe during the NSM-tuned coassembly process (Fig. 3E). While *J* aggregation is generally believed to enhance fluorescence, coassembly with embelin and isoquercetin trapped the probe completely in a dark state (Fig. 3F). This rare nonfluorescent *J* aggregates allows the probe to function as an indicator of responsive release both in vitro and in vivo (Fig. 3G).

We further investigated the driving forces underlying the supramolecular assembly. Increasing SDS concentrations not only recovered the Q band but also enhanced the fluorescence intensity of the probe (fig. S5). Consistent with these findings, the hydrodynamic diameter gradually increased beyond 300 nm, accompanied by noticeable heterogeneity (Fig. 3J). Urea (0.05 to 2 M) triggered rapid disassembly of TNPs, resulting in the formation of micron-sized aggregates (Fig. 3K). While 5% glucose solution showed no effect on self-assembly, saline and NaCl solutions (0.5 to 1 M) promoted the formation of uniform particles (~1.2 μm). Notably, 2 M NaCl induced heterogeneous disassembly (Fig. 3L). These results collectively demonstrate the involvement of diverse noncovalent interactions—including hydrophobic interactions, hydrogen bonding, and electrostatic interactions—in the self-assembly process. Given that noncovalent linkages are relatively weak and dynamic, supramolecular systems are often endowed with stimuli responsiveness. In vitro studies demonstrated a markedly faster release rate of TNPs under slightly acidic condition (pH 6.5) compared to physiological neutral condition (pH 7.4), which is attributed to the disruption of hydrogen bonds in the acidic environment (Fig. 3, H and I). Collectively, embelin and isoquercetin were successfully coassembled with ZnPc5K into supramolecular nanoparticles.

### In situ formation of PC on TNPs (TNP@PCs)

**Fig. 4.**
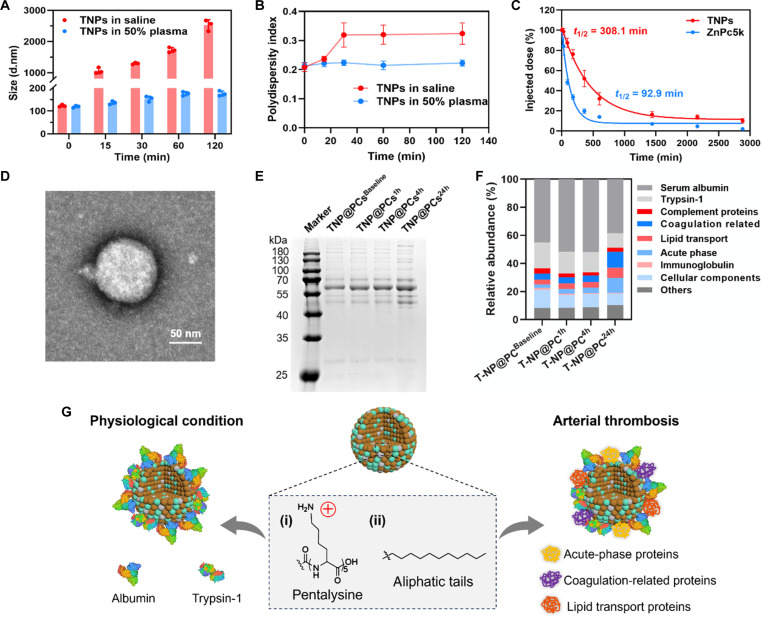
In situ formation of PC on TNPs (TNP@PCs). (**A**) Size and (**B**) PDI of TNPs stored in saline and 50% plasma at different time points. Data are means ± SD (*n* = 3 independent samples). (**C**) Systemic circulation time of TNPs and ZnPc5K after intravenous injection. Data are means ± SD (*n* = 4 independent samples). (**D**) Representative TEM image of TNP@PCs. Scale bar, 50 μm. (**E**) Qualitative analysis of proteins adsorbed on TNPs incubated with plasma from healthy mice and CAT mice at 1, 4, and 24 hours post-surgery by SDS-PAGE. (**F**) Relative abundances of coronal proteins on TNP@PCs^Baseline^, TNP@PCs^1h^, TNP@PCs^4h^, and TNP@PCs^24h^, classified by biological function. (**G**) Schematic diagram illustrating the distinction in plasma proteins attracted by TNPs with unique surface properties under physiological conditions and in the arterial thrombotic environment.

When stored in saline at room temperature, the hydrodynamic diameter of TNPs gradually increased to about 2.5 μm over a period of 2 hours. Notably, TNPs exhibited good colloidal stability in 50% plasma, with only slight increases in size and stable polydispersity index (PDI) (Fig. 4, A and B). To investigate the reasons behind the stability of TNPs in plasma, we established an in vitro device to mimic blood circulation (fig. S6A). When TNPs were circulated in 50% human plasma, the hydrodynamic diameter increased slightly, while the zeta potential shifted from positive to negative (fig. S6B). The retarded migration of protein bands, particularly serum albumin, and the retention of the probe in the gel, as evidenced by native–polyacrylamide gel electrophoresis (PAGE) analysis and subsequent fluorescence molecular tomography (FMT) imaging, further demonstrated the interaction between TNPs and plasma proteins (fig. S7). Consistent with these findings, ZnPc5K delivered in TNPs exhibited a significantly prolonged circulation time compared to an equivalent amount of ZnPc5K in saline. Notably, the circulation half-life of TNPs was 3.3 times longer than that of free ZnPc5K (Fig. 4C). When used as a probe for thrombus imaging, the positively charged pentalysine of ZnPc5K directly interacted with the negatively charged thrombi, facilitating targeted accumulation at the thrombus site. However, at the nanoscale, the exposed positive charges promote protein binding onto the surface of nanoparticles. We propose that the in situ PC camouflage enhances the stability of supramolecular assemblies in plasma. This shift in behavior highlights the context-dependent role of pentalysine, where molecular-scale electrostatic interactions facilitate thrombus targeting, while nanoscale surface interactions drive PC formation and stabilization.

The TEM image revealed clusters of plasma proteins on nanoparticles, giving the surfaces a mottled appearance (Fig. 4D). Considering that PC affects the in vivo fate of micro-/nanodelivery systems, we further characterize the components of PC and their changes in the pathophysiological environment (*34*–*36*). Bicinchoninic acid (BCA) analysis indicated that the total protein mass adsorbed on TNPs was similar when incubated with plasma from healthy mice and carotid artery thrombosis (CAT) mice at 1, 4, and 24 hours post-surgery (fig. S8). To profile the coronal proteins, we conducted SDS-PAGE analysis followed by liquid chromatography tandem mass spectrometry (LC-MS/MS) identification (Fig. 4E). The Venn diagram showed that 168, 165, 167, and 161 kinds of proteins were identified in the corona on TNP@PCs^Baseline^, TNP@PCs^1h^, TNP@PCs^4h^, and TNP@PCs^24h^, respectively. Notably, two exclusive coronal components found in TNP@PCs^24h^, BPIFA2 and vimentin, were associated with innate immune response and endothelial-mesenchymal transition (EndMT), respectively, indicating inflammation and excessive vascular remodeling after carotid artery injury (fig. S9A). Among the identified coronal proteins, serum albumin and tryspin-1 together accounted for 63.4% of the total protein mass on TNP@PCs^Baseline^, likely due to the exposure of pentalysine moiety and embelin’s aliphatic tail on the surface of TNPs. Other components were classified into categories such as coagulation-related proteins, immunoglobulins, lipid transport proteins, complement proteins, acute-phase proteins, cellular components, and others based on their physiological functions (Fig. 4F). The proportions of albumin and trypsin-1 decreased to 48.7%, while those of coagulation-related proteins, lipid transport proteins, and acute-phase proteins increased on TNP@PCs^24h^. The heatmap displayed the top 32 components with relative high abundances and significant changing trends (fig. S9B). Specifically, alpha-1-antitrypsin (AAT), apolipoprotein A-IV (ApoA4), apolipoprotein E (ApoE), and fibrinogen exhibited prominent upward trends, whereas complement C1q, keratin, and immunoglobulins showed opposite trends. Further signaling pathway analysis revealed that the protein entities with increasing abundances were involved in the platelet activation, signaling and aggregation, regulation of insulin-like growth factor transport and uptake, formation of fibrin clot, MAP2K and MAPK activation, and neutrophil degranulation, reflecting the pathological features of arterial thrombosis (fig. S9C) (*37*–*39*). These results indicate that TNPs exhibit a tendency to form PC in situ, with the composition influenced by both the surface properties and pathological environment (Fig. 4G).

### Site-specific cargo release of TNP@PCs under high shear stress

The supramolecular assembly process relies on a balance between driving and dispersive forces, which can typically be regulated through external stimuli, often enabling on-demand cargo release. In the context of arterial thrombosis, supramolecular assemblies are particularly influenced by the microenvironmental conditions, characterized by inflammation-induced acidity and elevated shear stress. Specifically, physiological shear stress in arteries typically ranges from 10 to 70 dyn/cm^2^, whereas under thrombotic conditions, it can exceed 100 dyn/cm^2^, with some regions reaching up to 1000 dyn/cm^2^ or higher due to vascular stenosis and hemodynamic alterations (*40*, *41*). Because Korin *et al*. (*41*) introduced mechanosensitive drug delivery systems in 2012, shear stress at the thrombus site have been used to trigger drug release (*42*, *43*). Recently, Lu *et al.* (*44*) demonstrated a gradual loss of precoated ApoE attachment on graphene/gold under high tumoral interstitial pressure. Besides the pH-triggered release shown in Fig. 3 (H and I), we hypothesize that the heightened mechanical stress in a thrombotic artery will disturb the PC on TNPs surface, as well as TNPs themselves, which are stabilized by noncovalent interactions.

**Fig. 5.**
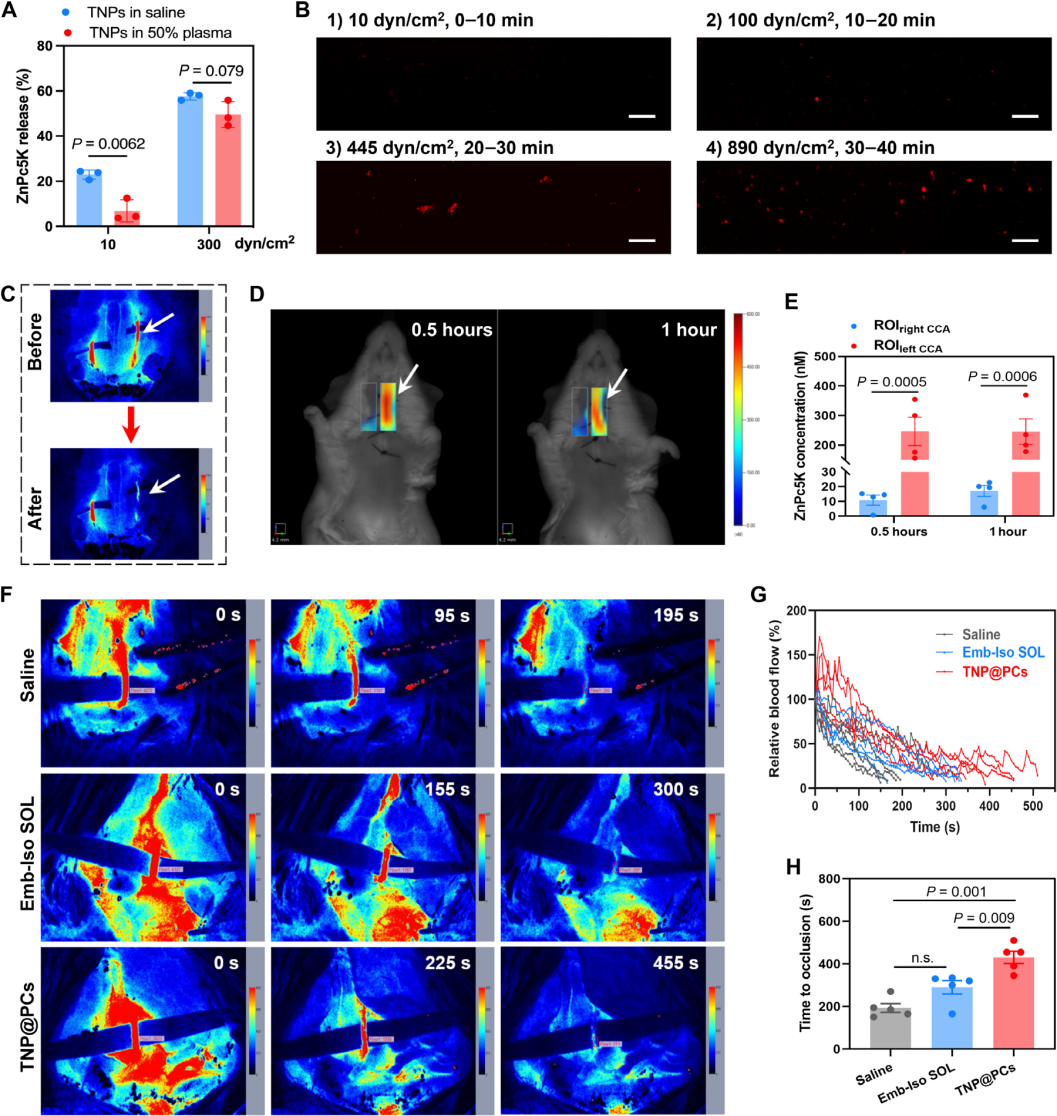
Site-specific cargo release of TNP@PCs under high shear stress. (**A**) ZnPc5K release from TNPs circulating in saline and 50% plasma for 15 min under different shear stress. Data are means ± SD (*n* = 3 independent samples). (**B**) Representative IVIS images showing cumulatively released ZnPc5K binding to fibrin film in μ-slide I^0.2^ chambers, from TNPs circulating in 50% plasma under different shear stress over a 40-min period. The images were captured at 10, 20, 30, and 40 min, respectively. Scale bars, 500 μm. (**C**) LSCI of CCA, showing blood flow in the left CCA decreased to about 10% of baseline after topical application of ferric chloride. (**D**) Representative FMT images of bilateral CCAs at 0.5 and 1 hour post-TNPs treatment. (**E**) Quantification of ZnPc5K concentrations in the left and right CCA. Data are means ± SEM (*n* = 4 mice). (**F**) Representative real-time LSCI of the left CCA post-ferric chloride challenge until rBF decreased to about 10% of baseline. Mice were pretreated with saline, Emb-Iso SOL, and TNPs intravenously 10 min before vascular injury. (**G**) Real-time blood flow dynamics in the left CCA normalized using blood flow before ferric chloride challenge as a standard. (**H**) Corresponding TTO in different groups. Data are means ± SEM (*n* = 5 mice). Statistical analysis was performed using unpaired Student’s *t* test for (A), one-way ANOVA with Tukey’s post hoc analysis for (H), and two-way ANOVA with Sidak’s post hoc analysis for (E). n.s., *P* > 0.05.

To test this hypothesis, we assessed the in vitro release performance of TNPs circulating in saline and 50% plasma, respectively. The flow rate and the resulting shear stress in the system were controlled by adjusting the pump speed. After circulating in saline under the normal range of arterial shear stress (10 dyn/cm^2^) for 15 min, the cumulative release of TNPs reached 22.9%. In contrast, the release of TNPs circulating in 50% plasma under the same shear stress was only 6.8%, indicating the stability of the nanoparticles with PC camouflage in the circulation. When shear stress increased to 300 dyn/cm^2^, the cumulative release of both TNPs and its PC counterpart rose to around 50 to 60% (Fig. 5A). Fluorescence imaging of ibidi μ-slide precoated with fibrin further indicated cargo release from TNP@PCs in a shear stress–dependent manner (Fig. 5B).

We further comprehensively investigated the release performance of TNP@PCs in vivo. The left carotid artery of mice was injured by topical application of ferric chloride, resulting in nearly 90% stenosis, and was followed by TNPs administration through tail vein (Fig. 5C). FMT imaging of bilateral carotid arteries simultaneously showed markedly enhanced fluorescence intensity in the left carotid artery at 0.5 and 1 hour compared with that in the contralateral artery (Fig. 5D). This result indicated the release of the probe at the thrombotic site, making thrombus visible. Further quantification demonstrated that the concentrations of the probe in the left carotid arteries were 22.8 and 14.4 times higher than those in the contralateral arteries at 0.5 and 1 hour, respectively (Fig. 5E). To exclude the possibility that the observed fluorescence enhancement in the left carotid arteries was due to targeting of the premature released probe to thrombus, we further investigated the impact of preadministration of TNPs on the time to occlusion (TTO) after carotid artery injury. Real-time monitoring of blood flow by laser speckle contrast imaging (LSCI) showed that preadministration of TNPs significantly delayed TTO compared with saline and embelin-isoquercetin solution (Emb-Iso SOL) groups (Fig. 5, F to H). Both in vitro and in vivo results strongly suggest that TNP@PCs with dual responsiveness undergo PC shedding and nanoparticle disassembly under pathological microenvironment, enabling thrombotic site-specific cargo release.

### Therapeutic effects of TNP@PCs on arterial thrombosis

**Fig. 6.**
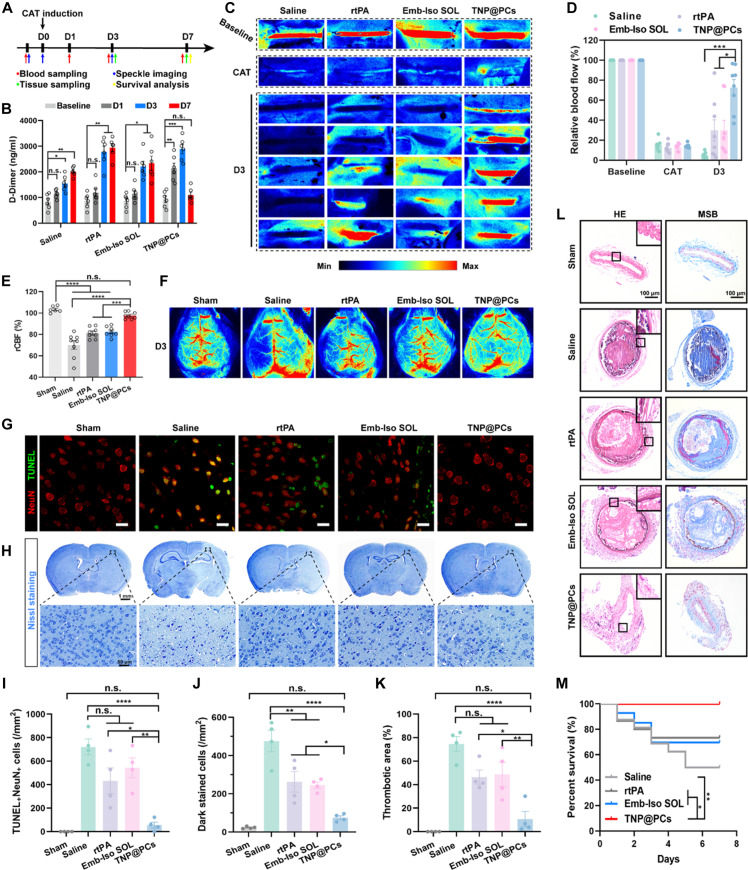
Therapeutic effects of TNP@PCs on CAT and subsequent chronic cerebral ischemia. (**A**) Schematic diagram of CAT induction, sampling and analysis at different time points. (**B**) Plasma D-dimer concentrations. Data were means ± SEM (*n* = 6 mice). (**C**) Representative LSCI of CCA before CAT induction, after CAT and on day 3. (**D**) Corresponding rBF normalized using blood flow before CAT as a standard. Data were means ± SEM (*n* = 8 mice). (**E**) rCBF and (**F**) representative LSCI of cerebral cortex on D3. Data were means ± SEM (*n* = 6 to 8 mice). (**G**) Representative TUNEL and (**H**) Nissl-stained brain tissues on D3. Scale bars, 20 μm in (G), 1 mm in the top of (H), and 50 μm in the bottom of (H). (**I**) Number of TUNEL^+^NeuN^+^ cells and (**J**) dark-stained neuronal cells per mm^2^ on D3. Data were means ± SEM (*n* = 4 mice). (**K**) Thrombotic areas of injured CCA on day 7. Data were means ± SEM (*n* = 3 mice). (**L**) Representative H&E and MSB-stained histological sections of injured CCA on day 7. Scale bars, 100 μm (*n* = 3 independent samples). (**M**) Survival profiles of mice in various treatment groups (*n* = 14 to 16 mice). Statistical analysis was performed using one-way ANOVA with Tukey’s post hoc analysis in (E) and (I) to (K), and two-way ANOVA with Tukey’s post hoc analysis in (B) and (D). n.s. *P* > 0.05, *P < 0.05, **P < 0.01, ***P < 0.001, and ****P < 0.0001.

Although rare in cerebrovascular pathology, symptomatic carotid thrombosis is associated with a high risk of recurrent cerebral ischemia in patients. The lack of a clear consensus on its management strategy leads to high morbidity and mortality (*45*, *46*). In CAT mice, we evaluated the therapeutic effects of various treatments on both carotid artery thrombus and subsequent chronic cerebral ischemia (Fig. 6A). Intravenous injection of ZnPc5K had no effects on thrombus formation, thrombolysis, and chronic cerebral ischemia (figs. S10 to S12). Plasma D-dimer concentrations in saline-treated mice on day 1 (D1) was found to be comparable to baseline levels but continued to rise until D7. Similar trends were observed in the rtPA- and Emb-Iso SOL-treated mice, suggesting ongoing endothelial dysfunction and hypercoagulability. Notably, TNP@PCs group showed significantly increased plasma D-dimer levels on D1, indicating enhanced fibrin degradation. The D-dimer levels further increased on D3 but returned to baseline on D7, suggesting a restored balance between coagulation and fibrinolysis after TNP@PCs treatment (Fig. 6B). LSCI measurements revealed that blood perfusion in the injured carotid artery of saline-treated CAT mice was almost completely obstructed on D3. The thrombolytic effects of rtPA or Emb-Iso SOL varied greatly among mice, showing limited improvement in such case of severe vessel injury with FeCl_3_. In contrast, TNP@PCs significantly increased relative blood flow compared to other groups (Fig. 6, C and D). In addition, varying degrees of ischemia in the cerebral cortex was observed in the saline, rtPA, and Emb-Iso SOL groups, whereas TNP@PCs effectively alleviated cerebral cortex ischemia (Fig. 6, E and F). TdT-mediated terminal deoxynucleotidyl transferase–mediated deoxyuridine triphosphate nick end labeling (TUNEL) and Nissl staining on D3 revealed numerous dead or dying neurons with pyknotic morphology and fewer Nissl bodies in the saline group. rtPA and Emb-Iso SOL partially mitigated neuronal damage. Consistent with LSCI findings, TNP@PCs significantly reduced the number of NeuN^+^TUNEL^+^ cells and dark-stained neurons with shrunken morphology, markedly improving cerebral ischemia outcomes (Fig. 6, G to J).

To assess long-term therapeutic effects on thrombotic disease management, we performed histological analysis of FFPE sections of injured carotid arteries on D7. Hematoxylin and eosin (H&E) staining showed that TNP@PCs more effectively promoted fibrinolysis and prevented reocclusion compared to saline, rtPA, and Emb-Iso SOL treatments (Fig. 6, K and L). Martius scarlet blue (MSB) staining revealed newly formed fibrin on the injured endothelium in partially recanalized carotid arteries following rtPA treatment, indicating a risk of recurrent thrombosis (Fig. 6L). Moreover, the internal elastic membrane, which was disrupted and straightened post-ferric chloride injury, was restored to a wavy structure in TNP@PCs group, indicating improved systolic function of the carotid artery (Fig. 6L). Consistent with these findings, TNP@PCs treatment visibly reduced fibrin deposition (fig. S13). In terms of survival, approximately 50% of saline-treated mice died within 7 days post-thrombus induction. Survival rates in the rtPA and Emb-Iso SOL groups were similar, with no deaths after D3. Notably, no mice in TNP@PCs group succumbed during the study (Fig. 6M).

### Prothrombotic microenvironment remodeling and vascular repair by TNP@PCs

**Fig. 7.**
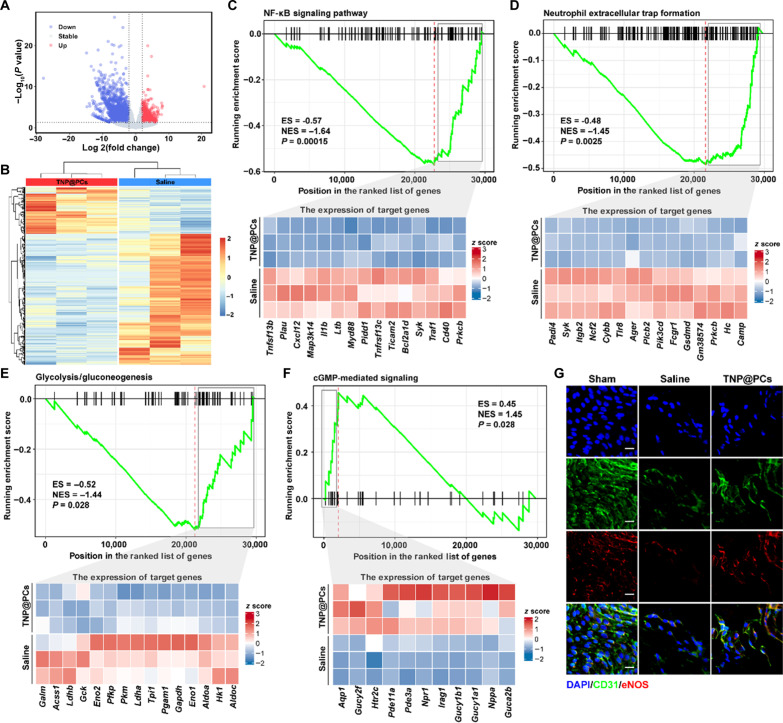
Therapeutic mechanism of TNP@PCs. (**A**) Volcano plots for DEGs between saline and TNP@PCs groups revealed by bulk RNA-seq analysis. (**B**) Hierarchical clustering analysis of DEGs. GSEA of injured CCA showing down-regulation of (**C**) NF-κB signaling, (**D**) NETs formation, (**E**) glycolysis/gluconeogenesis, and up-regulation of (**F**) cGMP-mediated signaling in TNP@PCs treated mice compared with those treated with saline and the correspondent heatmaps of the top 15 enriched genes. (**G**) Representative en face images of carotid arteries stained with anti-CD31, anti-eNOS, and DAPI. Scale bars, 20 μm (*n* = 3 independent samples).

We next investigated the underlying mechanisms responsible for TNP@PCs-mediated therapeutic effects on carotid artery thrombosis accompanied by chronic brain ischemia. A total of 2965 differentially expressed genes (DEGs) were identified in injured carotid arteries between saline and TNP@PCs groups on D3, with 778 genes up-regulated and 2187 genes down-regulated (Fig. 7A). Hierarchical clustering analysis of DEGs revealed a substantially different transcriptomic profile between the two groups (Fig. 7B).

Notably, in saline-treated mice, the expression of genes encoding PAI-1, IL-6, IL-1β, and TNF-α (*Serpine1*, *Il6*, *Il1b*, and *Tnf*) remained consistently elevated on D3. This finding aligns with immunofluorescence staining results presented in Fig. 2F, further demonstrating a PAI-1–dominated fibrinolysis-resistant state and an inflammatory microenvironment in the injured carotid arteries. In contrast, the transcription levels of these genes decreased following TNPs administration (fig. S14, A to D). To further explore the biological functions and major enrichment pathways of the DEGs, we used gene set enrichment analysis (GSEA)–based Gene Ontology (GO) enrichment and Kyoto Encyclopedia of Genes and Genomes (KEGG) analyses, which revealed significant down-regulation of NF-κB signaling, neutrophil extracellular traps (NETs) formation, and glycolysis in TNP@PCs group (Fig. 7, C to E). NF-κB signaling plays a crucial role in the pathogenesis of thrombotic diseases by promoting inflammation, endothelial activation and a procoagulant state. Persistent activation of NF-κB contributes to endothelial dysfunction, chemokines-driven neutrophil recruitment, and an increased likelihood of thrombus recurrence, worsening the prognosis of thrombotic diseases (*47*–*49*). TNP@PCs significantly inhibited NF-κB signaling and reduced chemokine secretion, preventing neutrophil recruitment and subsequent NETosis. One of the prominent targets of TNP@PCs against thrombosis involves glycolytic metabolism. Recent studies have shown that activated platelets convert their energy metabolism to glycolysis to adapt to the hypoxic and nutrient-deprived environment within dense clots (*50*, *51*). Considering the bleeding risk associated with current antiplatelet drugs, targeting metabolic regulatory mechanisms in platelets to prevent their activation represents a promising paradigm for combating arterial thrombosis (*52*). On the other hand, cGMP-mediated signaling, a well-studied protective pathway in ischemic diseases, was up-regulated in TNP@PCs group (Fig. 7F). The cGMP-mediated signaling pathway plays a crucial role in promoting vasodilation, reducing inflammation, and regulating coagulation and fibrinolysis system. Its up-regulation facilitates the improved homeostasis of injured blood vessels (*53*, *54*). The generation of cGMP was initiated by natriuretic peptides and nitric oxide (NO). Consistently, the mRNA level of endothelial NO synthase (eNOS or NOS3) was prominently up-regulated (fig. S14E). In addition, immunofluorescence imaging of eNOS and CD31 further demonstrated the repair of the arterial endothelium (Fig. 7G and fig. S15). Together, the notable down-regulation of NF-κB signaling, NETosis, and glycolysis, along with an up-regulation of cGMP-mediated signaling, indicates reduced inflammation and an improved metabolic process facilitated by TNP@PCs, thereby fostering antithrombotic and prorepair environment.

### Biosafety evaluation of TNP@PCs

Last, we investigated the biosafety of TNP@PCs to evaluate its potential for further translation. The cell counting kit-8 (CCK-8) assay demonstrated negligible cytotoxic effects of TNP@PCs on endothelial cells, even at concentrations of TNPs up to 320 μg/ml, indicating good cell compatibility (fig. S16A). When incubated with RBC suspension, the hemolysis rates of embelin, isoquercetin, and TNP@PCs at therapeutic doses were much lower than 5% (fig. S16B). In CAT mice, there were no significant differences in blood coagulation parameters—including activated partial thromboplastin time (APTT), prothrombin time (PT), tail bleeding time, and blood loss—in TNP@PCs group compared to saline. However, intravenous injection of rtPA led to significant changes in these parameters, suggesting a high risk of bleeding (fig. S17). We also tested the acute and chronic toxicity of TNP@PCs in healthy mice on day 4 and day 31, respectively. As expected, hematological indexes such as alanine transaminase and aspartate transaminase showed no significant variation after administering double doses of TNPs once daily for 3 days and a therapeutic dose every other day for 30 days (fig. S18). In addition, both short-term and long-term administration of TNPs caused no obvious histopathological changes in major organs, including brain, heart, liver, spleen, lung, and kidney, as demonstrated by H&E staining (fig. S19). Therefore, the in vitro and in vivo results support the notion that TNP@PCs exhibit satisfactory biosafety.

## DISCUSSION

In this study, we investigate the therapeutic potential and mechanism of a supramolecular nanoparticulate system targeting highly antifibrinolytic arterial thrombosis. Specifically, the supramolecular system codelivering bioregulators for the arterial thrombotic microenvironment is developed using a NSM-tuned self-assembly strategy. The resulting TNPs, with exposed positive charges and aliphatic tails, attract plasma proteins, primarily serum albumin—the major carrier protein for fatty acids in plasma forming a PC camouflage (TNP@PCs). This PC enhances the stability of the supramolecular system in physiological circulation. In the context of arterial thrombosis, however, plasma proteins associated with pathological conditions compete for binding to TNPs, resulting in alterations of PC composition. Under acidic conditions and pathological shear stress, TNP@PCs disassemble, effectively lighting up carotid thrombus and delaying carotid thromboembolism in vivo. Furthermore, TNP@PCs promote in situ carotid thrombus lysis and alleviate chronic cerebral ischemia by remodeling arterial thrombotic microenvironment.

Although rtPA is the first-line treatment for ischemic stroke and is widely used in the clinical management of acute myocardial infarction (AMI), substantial doses are required for effective thrombolytic therapy, likely due to elevated plasma levels of PAI-1. Proposed pharmacological strategies to enhance the therapeutic efficacy of rtPA include: (i) developing rtPA mutants with increased resistance to PAI-1, (ii) ultrasound-assisted thrombolytic therapy, and (iii) combining rtPA with antiplatelet or anticoagulant agents (*55*, *56*). However, these approaches still fall short of clinical needs, and arterial reocclusion following thrombolysis-primarily due to immunothrombosis remains a formidable challenge, underscoring the link between inflammation and thrombosis (*16*, *57*, *58*). In addition, combined antithrombotic therapy not only fails to completely prevent recurrent thrombotic events but also carriers a high risk of hemorrhagic transformation.

Damaged endothelial cells within a persistent prothrombotic pathological microenvironment hinder the recovery of vascular homeostasis. Dhanesha *et al.* (*48*) demonstrated that myeloid-specific integrin α9β1 exacerbates stoke following ischemia/reperfusion by promoting neutrophil recruitment, neutrophil-mediated platelet aggregation, and NETs formation. Furthermore, Griemert *et al.* (*59*) identified PAI-1–driven fibrinolysis resistance as a key feature of traumatic brain injury, further promoting the procoagulant phenotype transformation of cerebral microvessels and subsequent thrombosis. In this study, we use an active PAI-1–specific capture reagent, PAItrap, for enzyme-linked immunosorbent assay (ELISA) detection, revealing a significantly positive correlation between active PAI-1 and proinflammatory cytokine levels in the plasma of CAT mice. This finding highlights the importance of the coordinated regulation of uncontrolled inflammatory responses and excessive PAI-1 in remodeling the arterial thrombotic microenvironment. While PAI-1 and MPO-positive neutrophils are frequently colocated in clotted vessels, it remains unclear whether this indicates PAI-1 production by neutrophils or the binding of PAI-1 to neutrophils. In addition, we use a homemade rabbit serum polyclonal antibody for immunofluorescence detection of local PAI-1 levels in damaged vessels, which reflect total PAI-1 antigen levels rather than active PAI-1 levels. Nevertheless, bulk RNA sequencing (RNA-seq) analysis reveal a sustained increase in PAI-1 transcription levels in the injured carotid arteries on D3, supporting the PAI-1–dominated fibrinolytic resistance in the locally damaged vessels from a different perspective.

Supramolecular systems self-assembled from NSMs enable the regulation of microenvironments in various diseases (*21*). In this study, embelin, the principal PAI-1 inhibitor for arterial thrombotic microenvironment, is a phenolic lipid with poor water solubility. To achieve an effective and target-oriented development of supramolecular assemblies, we introduced an amphiphilic thrombus probe and used an NSM-tuned self-assembly strategy, resulting in TNPs with high drug loading capacity and an “off-on” fluorescence switch. A limitation of this strategy is the potential difference in the loading capacities of the two bioregulators within the supramolecular assemblies. Further investigation into the molecular assembly mechanism is needed to optimize codelivery efficiency.

Supramolecular assemblies based on noncovalent interactions are challenged by the complex environment in vivo. Despite the advances of polymer-coated and cell membrane–cloaked methods applying as the biomimetic camouflage over nanoplatforms, there are a few limitations that remain to be solved, such as biosafety and large-scale production. Moreover, once the camouflaged nanoplatforms enter the blood vessels, a PC will be attached to the surface of nanoparticles, which may counteract the expected effects of designed nanoplatforms. In this study, the surface of TNPs exhibits positive charges and aliphatic tails, preferentially binding fatty acid carrier proteins in plasma. The resulting PC ensures their stability in physiological circulation. This approach, which leverages the surface properties of supramolecular assemblies to attract plasma proteins and form a camouflaging PC, represents an innovative strategy to enhance stability within complex in vivo environments and prevent premature drug release. We also examine the influence of changes in plasma protein profiles under pathological conditions on the composition of coronal proteins. Significant differences are found in the PC composition on TNPs surface formed in mouse plasma before and after CAT. Notably, the proportion of albumin decrease in the PC formed 4 hours after thrombosis and is competitively replaced by coagulation-related proteins, lipid transport proteins, and acute-phase proteins. Specifically, AAT markedly increase in the PC 4 hours after thrombosis. Cruz *et al.* (*60*) demonstrated that surface decoration with an AAT-derived peptide motif enabled the specific anchorage of nanoparticles to activated neutrophils and platelet-neutrophil aggregates. Jonigk *et al.* (*61*) showed that AAT treatment exhibited anti-inflammatory and immunomodulatory properties in LPS-induced acute lung injury, reflected in significant reductions in infiltrating neutrophils. Strikingly, GSEA analysis in this study reveals significant inhibition of neutrophil recruitment and NETosis after TNP@PCs treatment. However, further research is needed to determine whether this therapeutic effect is partially attributed to increased AAT adsorption on the TNPs surface. TNP@PCs experience PC detachment and nanostructure disassembly under inflammation-induced acidic condition and sharply increased shear stress at arterial thrombotic sites, facilitating passive targeting and site-specific cargo release. In vivo, we visually observe the release of TNP@PCs at the thrombotic left carotid artery through FMT and demonstrate that on-demand release via pre-administration impedes carotid artery occlusion. In addition to inhibiting uncontrolled inflammation, the released bioregulators prevent excessive platelet activation and thrombus recurrence by inhibiting aerobic glycolysis and enhancing cGMP-mediated signaling.

For decades, the ultimate goal of thrombolytic and antithrombotic research has been to develop drugs that effectively balance efficacy and safety—a challenging endeavor due to the close relationship between hemostasis and thrombosis. TNP@PCs treatment remodels the prothrombotic microenvironment, promoting the repair of damaged endothelium. In conclusion, TNP@PCs demonstrated favorable biosafety, significantly increased survival rates, and improved outcomes in mice with CAT and chronic cerebral ischemia, representing a promising strategy for managing arterial thrombosis with high recurrence rates.

## MATERIALS AND METHODS

### Experimental design

This study investigated the feasibility of improving vascular homeostasis in the context of arterial thrombosis by synergistically inhibiting the cross-talk between PAI-1 and inflammation. This objective was addressed by (i) elucidating the presence of PAI-1 and inflammation levels in the microenvironment of arterial thrombosis and their correlation, (ii) developing and characterizing supramolecular assemblies codelivering bioregulators, (iii) characterizing in situ formation of PC at bio-nano interfaces and site-specific drug release, (iv) studying the therapeutic effects and mechanism of TNP@PCs on improving arterial thrombosis prognosis, and (v) evaluating the biosafety of TNP@PCs. Sample sizes were determined on the basis of prior experimental experience and review of the literature in the field. In vitro experiments were performed with at least three trials. For in vivo experiments, mice were randomized into treatment or control groups, and biological replicates are indicated in the figure legends. Animal allocation and data acquisition were performed in a blinded manner.

### Reagents

Embelin and isoquercetin were purchased from MedChemExpress. Pentalysine β-carbonyl phthalocyanine zinc (ZnPc5K) was synthesized as previously described. rtPA was purchased from Boehringer Ingelheim. Rabbit antisera against PAI-1–disu (point mutations of PAI-1 with very long half-life) were prepared by Nanjing Zoonbio Biotechnology. The BCA protein analysis kit was purchased from Solarbio. PAItrap and recombinant PAItrap-HSA fusion protein were expressed in *Pichia pastoris* (strain X33) and purified as previously reported (*62*).

### Human specimens

Human blood clots were retrieved from patients with cardioembolic stroke. Written informed consent was obtained from each participant under the Institutional Review Board (IRB)–approved protocol at Fujian Medical University Union Hospital [no. (2024) 256]. The collection of blood samples from healthy volunteers was approved by the IRB of Fujian Medical University Union Hospital, with informed consent obtained from all participating volunteers (no. 2024KY143).

### Animals

All animal studies were performed on adult male Institute of Cancer Research (ICR) mice aged 8 to 12 weeks and weighing 25 to 30 g. Mice were housed in a temperature- and humidity-controlled environment with a 12-hour light/12-hour dark cycle and free access to food and water. The experimentations were conducted in accordance with the Principles of Laboratory Animal Care of Fujian Medical University and approved by the Animal Ethics Committee of Fujian Medical University (IACUC FJMU 2024-Y-0194).

### Ferric chloride–induced mouse CAT

Mice were anesthetized via intraperitoneal injection of 1.25% tribromoethanol (Avertin). The common carotid artery (CCA), approximately 10 mm in length and situated below the bifurcation of the left external and internal carotid arteries, was meticulously isolated and exposed. A transparent, pliable plastic sheet was placed beneath the artery to separate it from adjacent blood vessels. Real-time blood flow (rBF) was monitored using the LSCI system (SIM BFI HR Pro; SIM Opto-Technology Co. Ltd). A 30 μl of 20% ferric chloride hexahydrate solution was applied until rBF decreased by approximately 90%. The carotid artery was then rinsed with saline, the plastic sheet was removed, and the neck incision was sutured. Sham-operated mice underwent anesthesia and artery exposure without topical application of ferric chloride.

### Immunostaining and fluorescence microscopy

FFPE sections of human blood clots were immunostained using a tyramine signal amplification (TSA) protocol. Briefly, sections were dewaxed, heat-retrieved in 10 mM tris/1 mM EDTA buffer (pH 9.0), and treated with 3% H_2_O_2_ for 30 min to inactivate endogenous peroxidase. After washing, the sections were blocked with 10% goat serum at 37°C for 30 min and incubated overnight at 4°C with rabbit antisera against PAI-1–disu (1:500). After washing with TBST, the sections were incubated with goat anti-rabbit IgG H&L (horseradish peroxidase) (Abcam, ab205718) at 37°C for 45 min. TSA marker iFluor 488–tyramide (AAT Bioquest, 11060) was then applied for 10 min at room temperature. After heating in the microwave oven to remove the primary and secondary antibodies, the sections were blocked with 10% donkey serum at 37°C for 30 min and incubated overnight at 4°C with rabbit anti-MPO (1:1000 dilution; Abcam, Ab208670). The sections were rinsed and incubated with Alexa Fluor 647 donkey anti-rabbit immunoglobulin G (H + L) (Abcam, ab150075). Last, the sections were sealed with antiquenching solution containing 4′,6-diamidino-2-phenylindole (DAPI) and observed using a digital slide scanner (Pannoramic DESK, P-MIDI, P250, P1000; 3D HISTECH).

For en face preparation of CCAs, the exposed hearts of anesthetized mice were perfused with precold PBS until the effluent ran clear, followed by 4% paraformaldehyde in PBS. The left carotid arteries were harvested and placed in fixative on ice. Under a stereomicroscope, the arteries were transferred to a petri dish lined with dissecting wax, and connective tissues were carefully removed. The arteries were longitudinally separated and opened to expose the endothelium. After antigen retrieval [100 mM tris and 5% urea (pH 9.5)], the vessels were transferred to separate wells in a 12-well plate for immunostaining.

Frozen sections (10 μm) or en face preparations of CCAs were blocked with 10% goat serum containing 0.3% Triton X-100 for 2 hours. The samples were then stained overnight at 4°C with following primary antibodies: rabbit antisera against PAI-1–disu (1:500), mouse anti-MPO (1:500; Servicebio, GB12224), rabbit anti-CD31 (1:200; Servicebio, GB113151), mouse anti-CD31 (1:50; Abmart, MU17118), rabbit anti-eNOS (1:50; Abmart, PS02827S). After washing with phosphate buffered saline with tween 20 (PBST), the samples were incubated with Alexa Fluor 488, Alexa Fluor 594, or Cy3-conjugated secondary antibodies for 1 hour at 37°C (Thermo Fisher Scientific). Nuclei were counterstained with DAPI, and fluorescent images were acquired using confocal microscopy (FV1000, Olympus).

### Enzyme-linked immunosorbent assay

The concentrations of IL-6, TNF-α, IL-1β, and D-dimer in plasma samples from mice were measured using ELISA kits (Elabscience). The active form of PAI-1 was detected using a specialized ELISA method based on a highly specific trapping agent (PAItrap-HSA), which was previously developed (*62*).

### Preparation and characterization of TNPs

TNPs were prepared by a nanoprecipitation method as described in previous reports (*63*). Briefly, 4 ml of DI water was added to a mixture containing 10 μl of ZnPc5K (10 mM), 10 μl of isoquercetin (10 mM), and 40 μl of embelin (10 mM) under mild stirring for 15 min. After standing at room temperature for 1 hour, the mixture was centrifuged at 1200 rpm for 15 min to remove large aggregates. The supernatant was then centrifuged at 14,500 rpm for 30 min to remove free drugs, and the deposit was resuspended.

The appearance of TNPs was observed using a TEM (JEM-1200 EX, Japan). The particle size distribution and zeta potential of TNPs were measured using a Zetasizer Nano ZS analyzer (Malvern, UK). The absorption and fluorescence spectrum were recorded using a microplate reader (Synergy H1, BioTek, USA). The content of ZnPc5K was determined using UV-vis spectroscopy, and the contents of isoquercetin and embelin were measured by high-performance liquid chromatography (HPLC) with a C18 column. To investigate the intermolecular forces in the self-assembly, TNPs were incubated with different concentrations of SDS, urea, and NaCl for 30 min, respectively. After incubation, the size, PDI, absorption, and fluorescence spectra were detected.

In vitro pH-triggered drug release was studied by dialysis method. TNPs (1 ml) were added to a dialysis bag (MWCO = 10 kDa) and immersed in 30 ml of PBS containing 1% (w/v) Tween 80 (pH 7.4 or 6.5) at 37°C with a constant stirring at 75 rpm. At predetermined time points, 1 ml of the release medium was collected and replaced with an equal volume of fresh medium. Embelin concentrations were determined by HPLC. Stability of TNPs in physiological fluids, i.e., saline and 50% plasma, was studied. The hydrodynamic diameter and PDI variations of TNPs over the course of 2 hours were detected by DLS.

To determine the circulating half-lives, healthy mice were randomly divided into two groups and intravenously administered TNPs or an equivalent amount of ZnPc5K via tail vein injection (*n* = 4 per group). At predetermined time points, 100 μl of blood was collected from the retro-orbital venous plexus. The blood samples were centrifuged at 1200 rpm for 10 min, and 45 μl of supernatant was collected. Subsequently, 5 μl of 10% SDS solution was added to the supernatant and thoroughly mixed. Fluorescence intensity was measured using a microplate reader at excitation/emission wavelengths of 610 nm/690 nm. The amount of ZnPc5K (in % of injected dose) as a function of times was plotted.

### Proteomic identification of coronal proteins

TNPs were incubated with plasma samples at 37°C for 1 hour, and TNP@PCs were collected by centrifugation at 14500 rpm for 30 min. For native-PAGE assays, the sediment was resuspended and loaded onto a 12% native-PAGE gel. Electrophoresis was performed in tris-glycine buffer at 120 V for 3 hours. Recombinant HSA, plasma, and TNPs alone were also assessed as controls.

SDS-PAGE and LC-MS/MS were performed to identify proteins adsorbed on TNPs incubated with plasma samples from CAT mice at 0, 1, 4, and 24 hours post-surgery. BCA assay was used to quantify the protein concentrations (Bio-Rad). Then, the protein extracts were separated in a 12% SDS-PAGE gel under a constant voltage of 180 V for 1 hour. Last, coomassie brilliant blue (CBB)-stained protein bands were visualized using Bio-Rad ChemiDoc imaging system. The protein bands were then cut from the gel and underwent reduction by 100 mM DTT, alkylation by 200 mM iodoacetic acid (IAA), and trypsinization at 37°C overnight. The protein samples were subsequently desalted and lyophilized. Last, the protein samples were resuspended in 0.1% formic acid and analyzed by LC-MS/MS.

### In vitro shear stress–induced cargo release

An in vitro circulation simulation system was developed, consisting of a reservoir, a μ-slide I flow chamber (ibidi, Germany), and a peristaltic pump. Shear stress within the slide was regulated by adjusting the flow rate and calculated using the formula: τ = 4μ*Q*/π*r*^3^, where μ represents fluid viscosity, *Q* is the flow rate, and *r* is the tube radius.

TNPs (33.3 μg/ml, 3 ml) in saline or plasma (diluted 1:1 in saline to obtain 50% plasma) were circulated through the μ-slide chamber under shear stress of 10 and 300 dyn/cm^2^, respectively. After 15 min, intact nanoparticles were collected through high-speed centrifugation. The fluorescent intensity was measured after dissolving the remaining TNPs in DMSO and normalized to the total amount. For studies on the release and binding of the probe to clots, fibrinogen (150 μg/ml) was first added to the μ-slide chamber, followed by thrombin (0.2 U/ml). The mixture was gently mixed and incubated at 37°C for 1 hour to allow in situ fibrin clot formation. Subsequently, TNPs in 50% plasma (33.3 μg/ml, 3 ml) were circulated under shear stress ranging from 10 to 890 dyn/cm^2^ for a period of 40 min, covering both physiological and pathological shear conditions (*42*, *64*). Fluorescence microscopic images were captured using a stereo fluorescence microscope.

### In vivo site-specific cargo release

In vivo targeted delivery of TNP@PCs and site-specific ZnPc5K release were visualized using FMT 2500TM LX instrument (PerkinElmer, USA). A 680-nm laser diode was used to excite ZnPc5K monomer, and fluorescence emission at 690 to 740 nm was detected. Three-dimensional reconstruction was carried out, and the quantification of ZnPc5K concentrations was provided by the software TrueQuant v3.0 (PerkinElmer, USA).

In addition, the effect of site-specific bioregulators release on TTO of damaged carotid artery was investigated by preadministration of TNPs. Mice were randomly divided into three groups and intravenously injected with saline, Emb-Iso SOL [embelin (2 μmol/kg) and isoquercetin (80 nmol/kg) in saline], and TNPs (1 mg/kg), respectively. After 10 min, the left CCA was exposed and socked in 20% ferric chloride hexahydrate. The blood flow was monitored in real time by LSCI and recorded every 5 s until the blood flow drops to about 10% of the baseline.

### Thrombosis treatment and prevention

Mice were randomized to different groups for the treatments (Sham or CAT) and then further randomized into the following treatment groups: saline, rtPA (at a single dose of 10 mg/kg), Emb-Iso SOL [embelin (2 μmol/kg) and isoquercetin (80 nmol/kg) daily for 3 days], and TNPs (1 mg/kg daily for 3 days). Thrombolytic therapies were delivered intravenously via tail vein 10 min after CAT. To prevent starvation after surgery, jelly food was provided in a petri dish on the cage floor. The survival status of mice was monitored, and the cumulative survival rates were recorded over a 7-day period.

Thrombotic area of injured CCA was evaluated using H&E staining. Fibrin staining was processed using modified MSB method (Solarbio). For immunohistochemistry, antigen retrieval was performed in EDTA buffer (pH 9.0), and slides were stained with anti-fibrin antibody (59D8, 4.2 μg/ml). Coronal FFPE sections (6 μm) were prepared from murine brain between 0.25 and −2.75 mm from bregma. For Nissl staining, slides were incubated in 1% Cresyl Violet for 15 min, followed by washing, dehydration, and mounting with dibutylphthalate polystyrene xylene (DPX). For neuronal apoptosis analysis, TUNEL was performed after NeuN immunofluorescence staining using a commercially available kit (Servicebio). Images were analyzed using ImageJ software. Data were expressed as mean number of cells/mm^2^.

### Transcriptome sequencing and bioinformatic analysis

Total RNA was extracted from injured CCA of mice treated with saline or TNP@PCs using TRIzol according to the manufacturer’s protocol and purified by ethanol precipitation. RNA-seq library construction and high-throughput sequencing were performed by Seqhealth Technology Co., LTD (Wuhan, China). Data processing and visualization were carried out using the R programming language (version 4.4.0) and the relevant R packages. DESeq2 (version 1.44.0) was used to identify DEGs between the two groups using the criteria: log_2_fold change ≥ 2 and *P* value < 0.05. Hierarchical clustering analysis of DEGs based on Pearson’s correlation distance was performed using the pheatmap package. GO and KEGG enrichment analysis based on GSEA were conducted using the gseGO and gseKEGG functions in the clusterProfiler package (version 4.12.0), respectively. *Mus musculus* genome information was annotated using the org.Mm.eg.db package. Normalized enrichment score was used to quantify enrichment magnitude, with a *P* value < 0.05 considered significant.

### Biosafety evaluation

The in vitro cytotoxicity of TNPs was examined on EA.hy926 cells, which were obtained from American Type Culture Collection (Rockville) and cultured in Dulbecco’s modified Eagle’s medium supplemented with 10% fetal bovine serum (Gibco BRL) at 37°C containing 5% CO_2_. Briefly, cells were seeded in 96-well plates at 10^4^ cells per well overnight, after which the culture media were replaced with fresh media containing TNPs at different concentrations. After 24 hours, 10 μl of CCK-8 was added into the media and incubated for 2 hours. The absorbance at 450 nm was subsequently measured using a microplate reader.

For hemolysis evaluation, fresh human whole blood was collected from healthy volunteers in 3.2% citrate (volume ratio of 9:1) and washed with saline to collect erythrocytes. Erythrocytes (2% ) suspension (v/v) was prepared and incubated with different treatments for 1 hour at 37°C. After centrifugation at 3000 rpm for 10 min, the supernatant was collected, and hemoglobin release was measured at 540 nm. Saline and deionized water were used as the negative and the positive controls, respectively. The hemolysis rate was calculated using the equation: *hemolysis* (%) = (*A*_*sample*_ − *A*_*negative control*_)/(*A*_*positive control*_ − *A*_*negative control*_) × 100%.

Indicators of extrinsic and endogenous pathways of coagulation (PT and APTT) in the plasma were analyzed 1-hour post-treatment using an automated coagulation analyzer (Rayto). For tail bleeding assay, 0.5 cm of the tail of CAT mice was cut 1 hour post-treatment and immersed in prewarmed saline at 37°C. Bleeding time was recorded until hemostasis was maintained for 10 s. Blood loss was quantified by measuring the absorbance at 575 nm. Sham-operated mice were used as negative controls. For acute toxicity evaluation, healthy ICR mice were treated with saline or double doses of TNPs (2 mg/kg) daily for 3 days. For chronic toxicity evaluation, mice were treated with saline or a standard dose of TNPs (1 mg/kg) every other day for 30 days. Serum samples and major organs were collected on the 4th day and 31th day for blood biochemical analysis and H&E staining, respectively.

### Statistical analysis

Data were analyzed using unpaired Student’s *t* test or analysis of variance (ANOVA), as indicated in the figure legends. Differences in survival were assessed by the Mantel-Cox log-rank test. A *P* value of <0.05 was considered statistically significant.

## Supplementary Materials

### The PDF file includes:

Figs. S1 to S19 Legends for datasets S1 to S6

### Other Supplementary Material for this manuscript includes the following:

Datasets S1 to S6
