# Supplementary material for: In situ protein corona–camouflaged supramolecular assemblies remodel thrombotic microenvironment for improved arterial homeostasis
Source: Sci Adv. 2025 May 2;11(18):eadu6676. doi: 10.1126/sciadv.adu6676 (PMC12047436; doi:10.1126/sciadv.adu6676)
Supplement: Supplementary file 1 — Figs. S1 to S19 Legends for datasets S1 to S6 [file sciadv.adu6676_sm.pdf]

Science Advances
AAAS

Supplementary Materials for

In situ protein corona–camouflaged supramolecular assemblies remodel thrombotic microenvironment for improved arterial homeostasis

Dan Chen *et al.*

Corresponding author: Mingdong Huang, HMD_lab@fzu.edu.cn; Zhaoyang Chen, chenzhaoyang888@126.com

Sci. Adv. **11**, eadu6676 (2025)
DOI: 10.1126/sciadv.adu6676

The PDF file includes:

Figs. S1 to S19
Legends for datasets S1 to S6

Other Supplementary Material for this manuscript includes the following:

Datasets S1 to S6

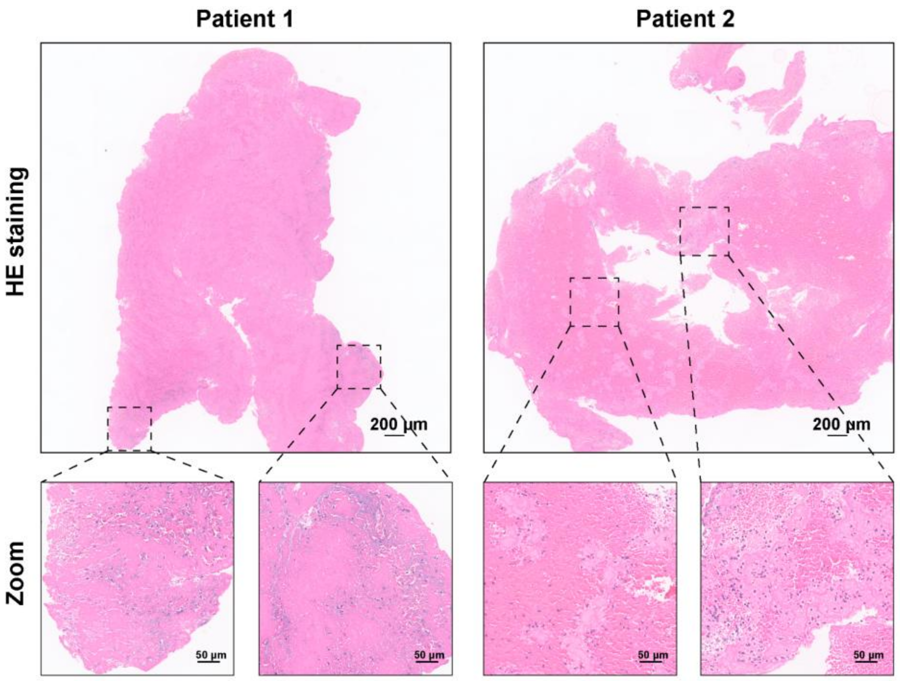

Fig. S1 Histologically processed and H&E-stained FFPE sections of well-organized clots retrieved from two female patients with cardiogenic stroke (Patient 1: 54 years old; Patient 2: 80 years old). A mass of interspersed leukocytes accumulated within the clots. Specifically, the clot from Patient 2 was aged and platelet-rich, with the influx of leukocytes particularly localized near the platelet-rich area.

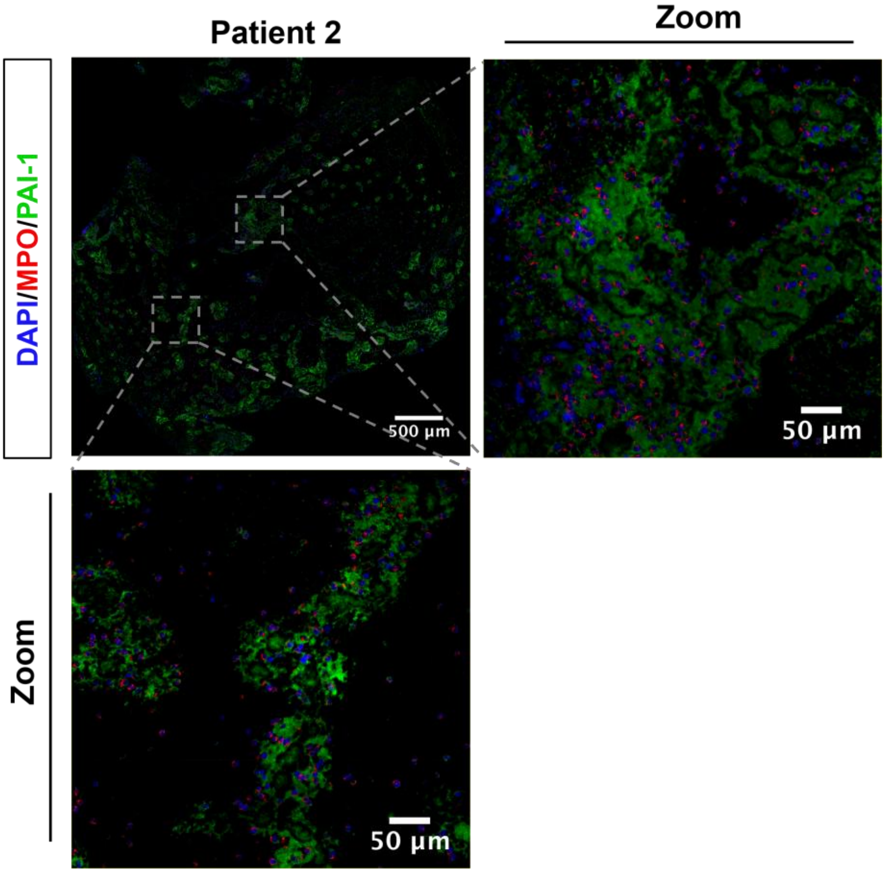

Fig. S2 Representative immunofluorescent images of clots from patient 2. FFPE sections were immunostained for PAI-1 and MPO, and the nuclei were counterstained with DAPI. Remarkable PAI-1 antigen was observed in the areas with abundant MPO-positive cells.

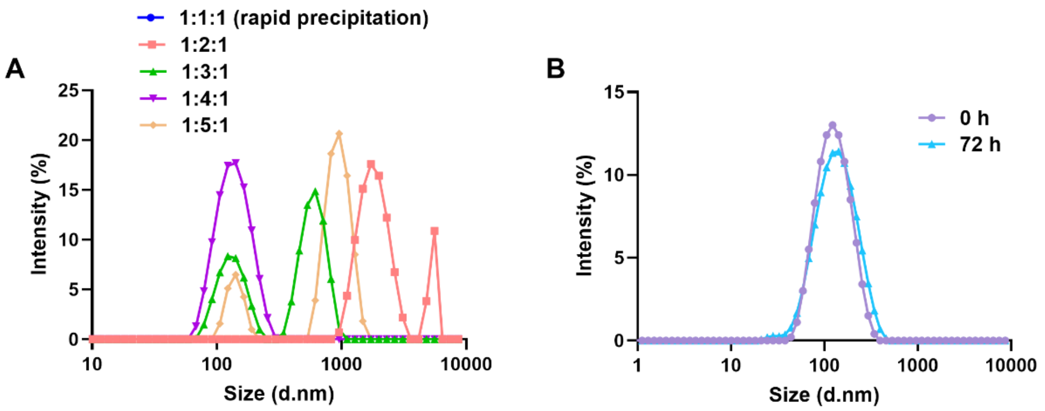

Fig. S3 Screening the optimal drug ratios in the self-assembly process. (**A**) Size distribution of the formulations with different ratios of ZnPc5K/embelin/isoquercetin (1:1:1 to 1:5:1). (**B**) Size distribution of TNPs with a ratio of 1:4:1 (ZnPc5K/embelin/isoquercetin) at room temperature over a period of 72 hours.

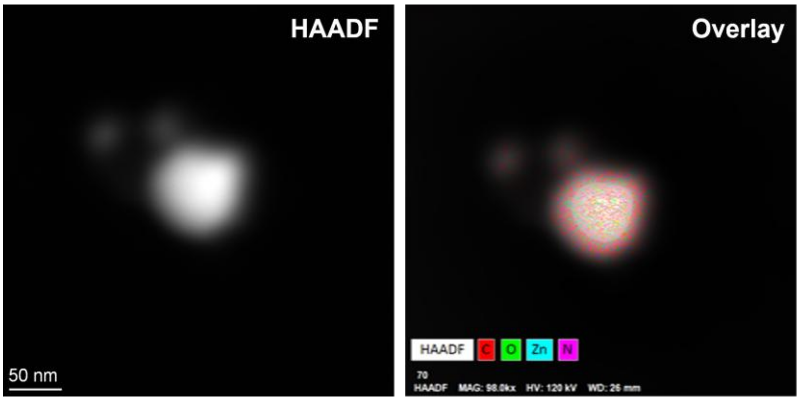

Fig. S4 Representative high-angle annular dark-field (HAADF) imaging and the overlay of EDS elemental mapping of TNPs (n = 2 independent samples).

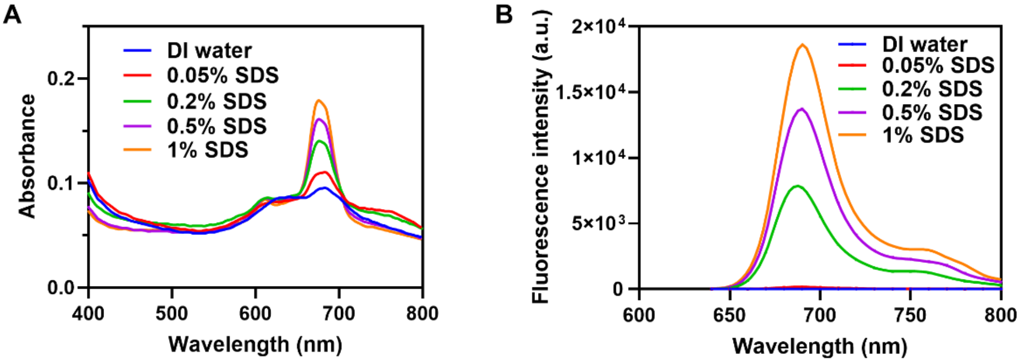

Fig. S5 The effect of SDS on TNPs self-assembly. (**A**) UV-vis absorption and (**B**) fluorescence spectrum of TNPs incubated in 0.05%~1% SDS.

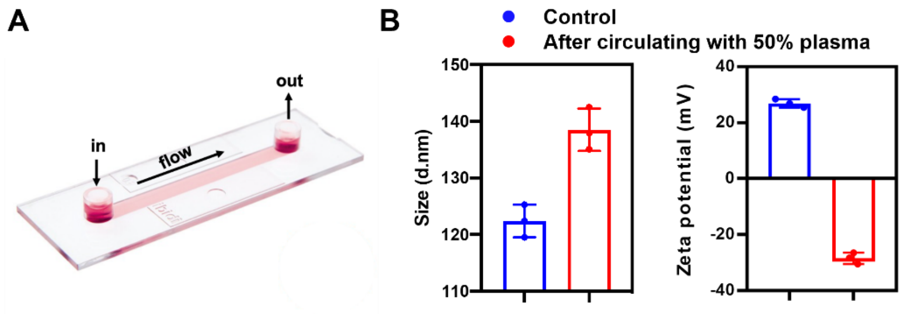

Fig. S6 *In vitro* evaluation of the stability of TNPs in plasma. (**A**) A microfluidic device to simulate the environment of arterial thrombosis with high shear stress. (**B**) Hydrodynamic diameter and zeta potential of TNPs and those circulated with 50% plasma (τ = 10 dyn/cm^2). Data are mean ± SD (n = 3 independent samples).

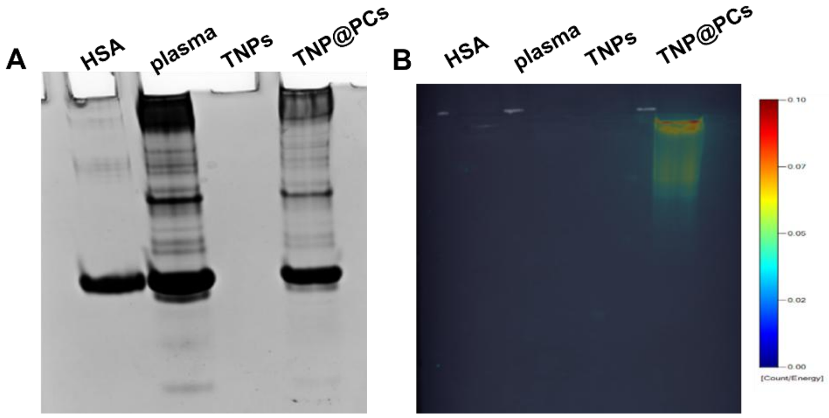

Fig. S7 Preliminary qualitative evaluation of the interaction between TNPs and plasma proteins. (**A**) Representative image of Native-PAGE gels with Coomassie Brilliant Blue staining of human serum albumin (HSA), plasma, TNPs, and TNP@PCs (TNPs were incubated with 50% plasma, collected by high-speed centrifugation and resuspended for analysis) (n = 3 independent experiments). (**B**) Representative FMT image of the gel in panel (**A**) (λ_{ex} = 680 nm).

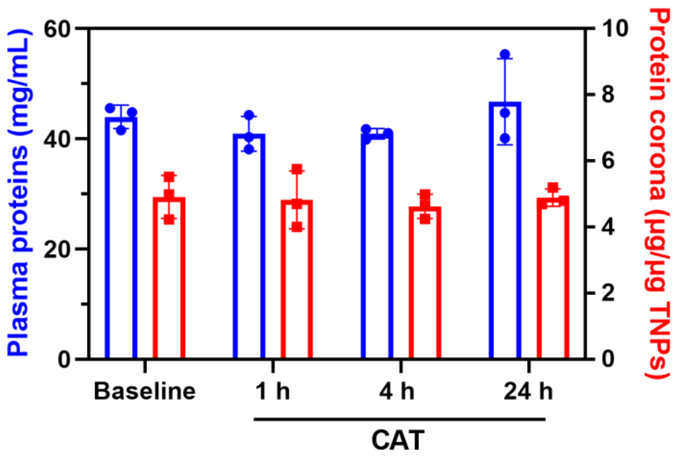

Fig. S8 Quantification of total plasma proteins and those adsorbed onto TNPs surface determined by BCA assay. Data are mean ± SD (n = 3 independent samples).

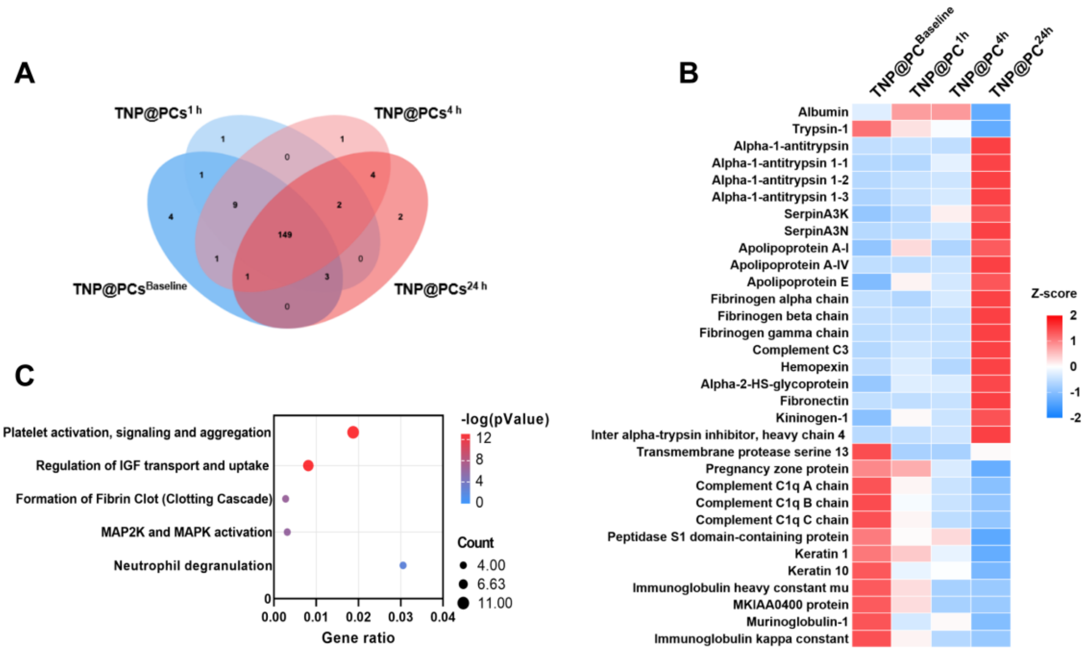

Fig. S9 Identification of coronal proteins by LC-MS/MS. (**A**) Venn diagram of identified proteins on TNP@PCs^{Baseline}, TNP@PCs^{1h}, TNP@PCs^{4h}, and TNP@PCs^{24h}. (**B**) Heat map of the top 32 coronal proteins with high relative abundancies and changing trends. (**C**) Reactome analysis of signaling pathways for the proteins in (**B**) with upward trends.

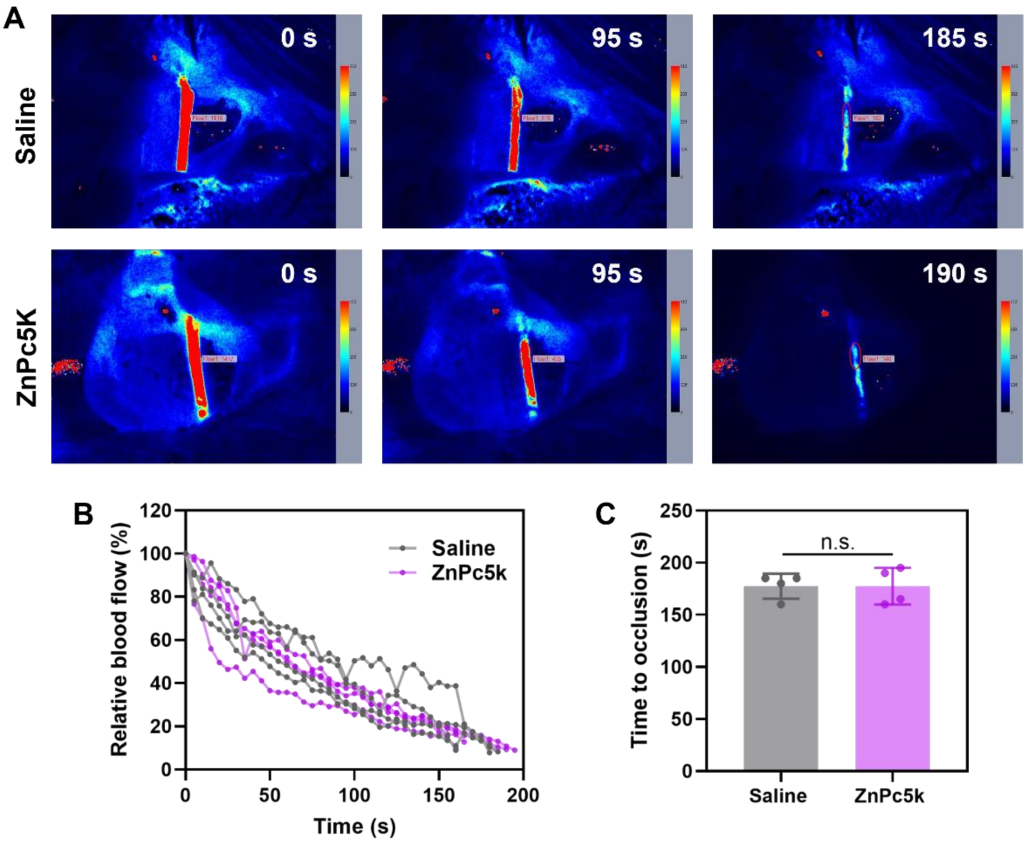

Fig. S10 The effect of ZnPc5K on thrombus formation. (**A**) Representative real-time LSCI of the left CCA post-ferric chloride challenge until rBF decreased to about 10% of baseline. Mice were pre-treated with saline and ZnPc5K (0.27 μmol/kg) intravenously 10 min before vascular injury. (**B**) Real-time blood flow dynamics in the left CCA normalized using blood flow before ferric chloride challenge as a standard. (**C**) Corresponding TTO in the two groups. Data are means ± SEM (n = 4 mice). Statistical analysis was performed using unpaired Student's t-test. [n.s.] $p > 0.05$.

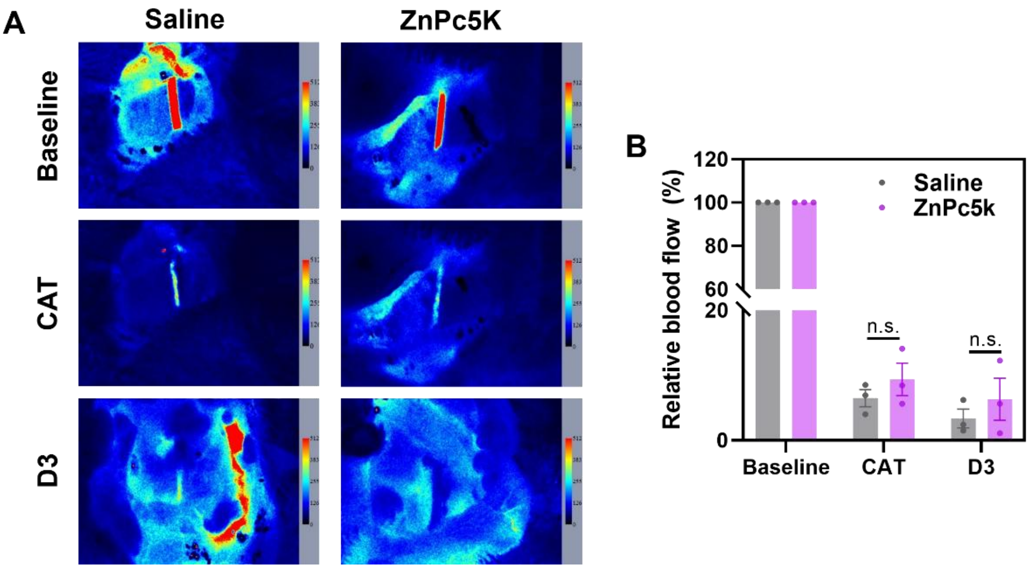

Fig. S11 The effect of ZnPc5K on thrombus dissolution. (**A**) Representative LSCI of CCA before CAT induction, after CAT and on day 3 after treatment with saline and ZnPc5K (0.27 μmol/kg daily for 3 days). (**B**) Corresponding rBF normalized using blood flow before CAT as a standard. Data were mean ± SEM (n = 3 mice). Statistical analysis was performed using two-way ANOVA with Sidak's post hoc analysis. $^{n.s.}$ $p > 0.05$.

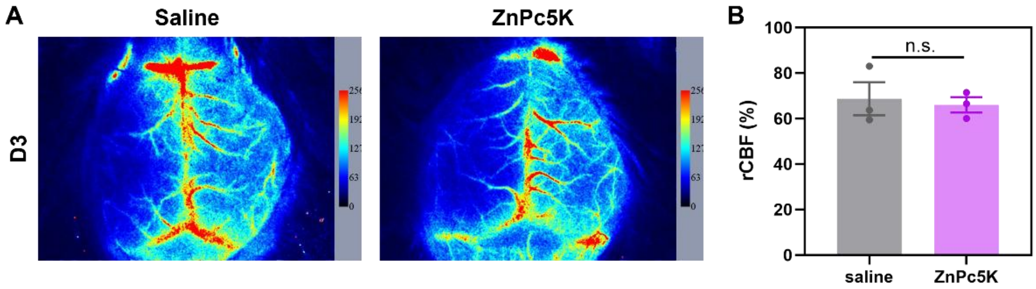

Fig. S12 The effect of ZnPc5K on chronic cerebral ischemia after CAT. (**A**) Representative LSCI of cerebral cortex and (**B**) the corresponding rCBF of CAT mice on day 3 after treatment with saline and ZnPc5K (0.27 μmol/kg daily for 3 days). Data were mean ± SEM (n = 3 mice). Statistical analysis was performed using unpaired Student's t-test. $^{n.s.}$ $p > 0.05$.

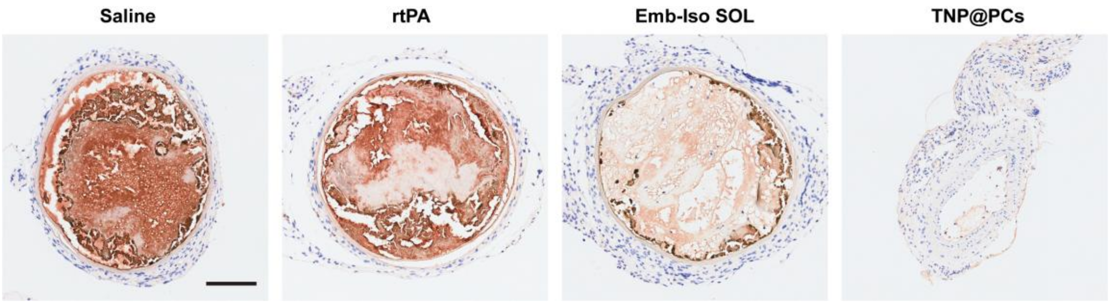

Fig. S13 Representative immunohistochemistry (IHC) images of fibrin (dark staining) on sections of CCA from CAT treated with different therapies on day 7. Scale bars: 100 μm (n = 2 independent samples).

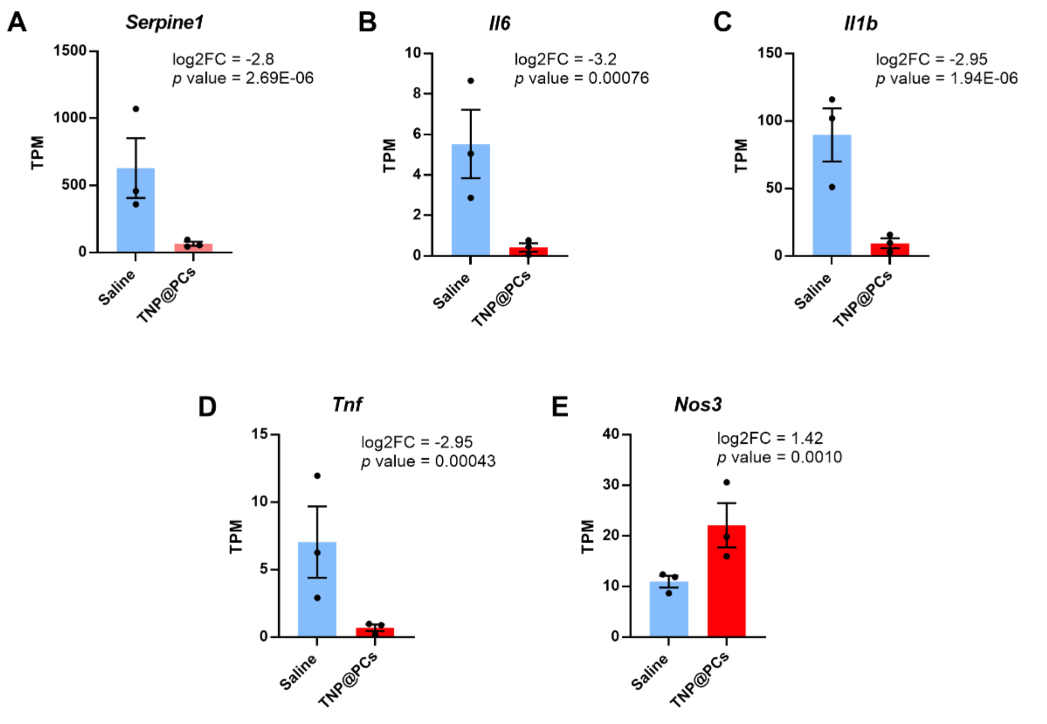

Fig. S14 The transcripts per million (TPM) of five key genes. Quantitation of (**A**) *Serpine1,* (**B**) *Il6,* (**C**) *Il1b,* (**D**) *Tnf,* and (**E**) *Nos3* TPM in saline and TNP@PCs groups. Log_2 fold change (log2FC) and *p* values were shown. Data were mean ± SEM (n = 3 independent samples).

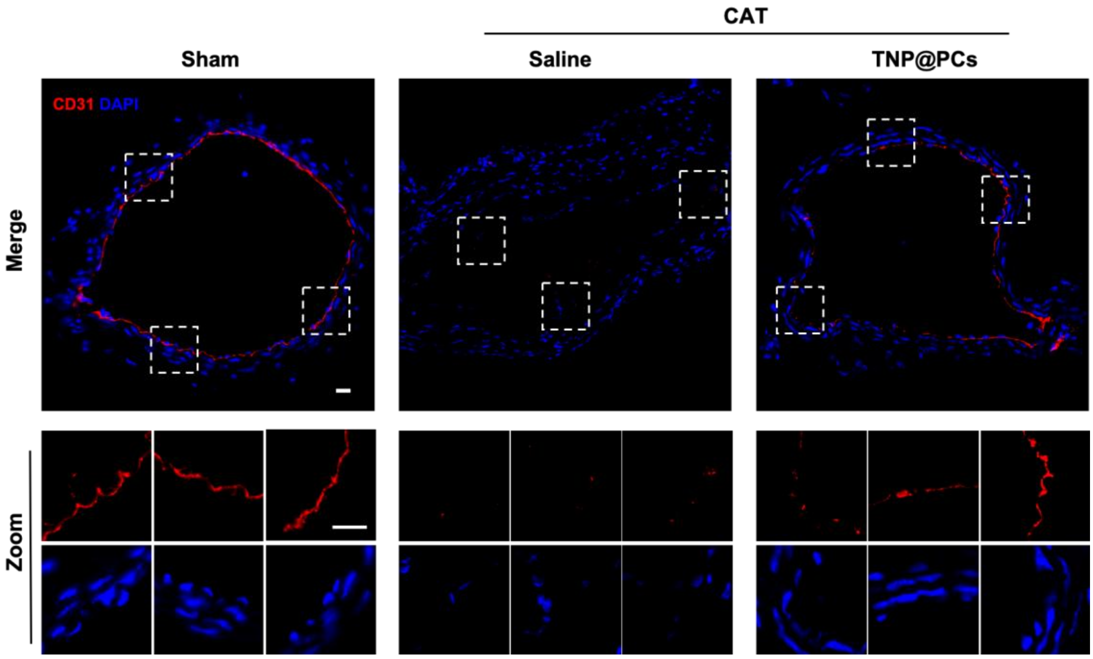

Fig. S15 Representative images of immunofluorescence stained cryosections of carotid arteries from sham mice, saline-treated CAT mice and TNPs-treated CAT mice on day 7. Red: CD31; Blue: DAPI. Scale bars: 20 μm (n = 3 independent samples).

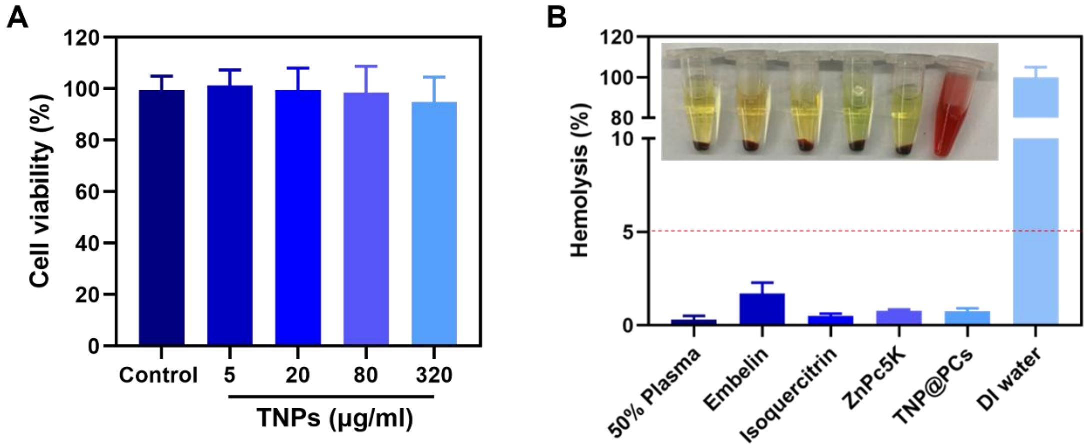

Fig. S16 ***In vitro*** **biosafety evaluation.** (**A**) Viability of endothelial cells after incubation with various concentrations of TNPs for 24 hours (n = 5 independent samples). (**B**) Hemolysis of human RBCs after incubation for 1 hour with 50% plasma, 50% plasma containing different drugs (70 μM embelin, 2.5 μM isoquercetrin, 10 μM ZnPc5K, and 33.6 μg/mL TNPs, respectively), and DI water. Data were mean ± SD (n = 3 independent samples).

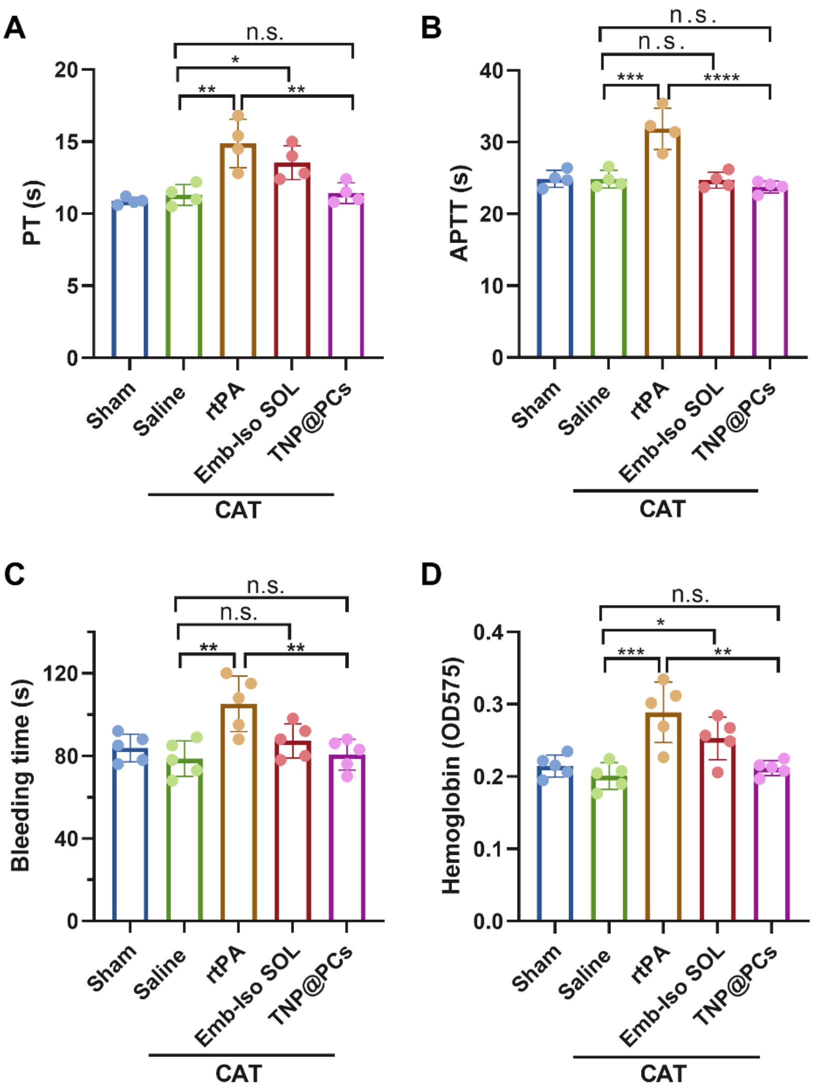

Fig. S17 *In vivo* assessment of coagulation and bleeding parameters. (**A**) PT and (**B**) APTT in the plasma of CAT mice 1-hour post-treatment with saline, rtPA, Emb-Iso SOL, and TNP@PCs. Data were mean ± SD (n = 4 independent samples). (**C**) Bleeding time and (**D**) blood loss of CAT mice treated with the indicated formulations. Data were mean ± SD (n = 5 independent samples). Statistical analysis was performed using one-way ANOVA with Tukey's post hoc analysis. $^{n.s.}\ p > 0.05$, $^{*}\ p < 0.05$, $^{**}\ p < 0.01$, $^{***}\ p < 0.001$, $^{****}\ p < 0.0001$.

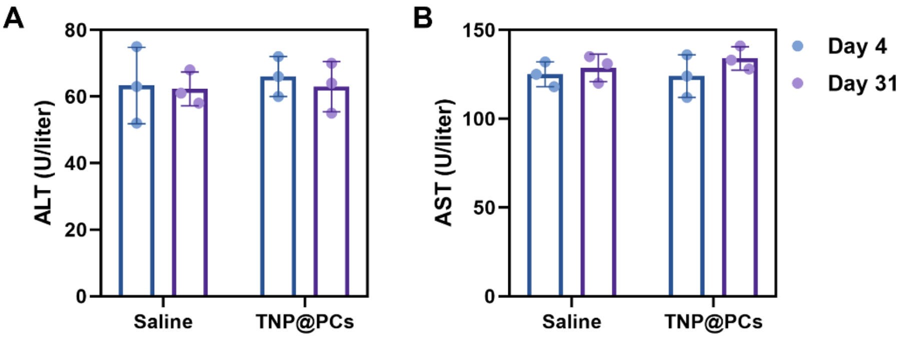

Fig. S18 Evaluation of liver function in acute and chronic toxicity studies. Serum levels of (**A**) ALT and (**B**) AST of healthy mice in acute (treated with TNPs at double dose daily for 3 days) and chronic toxicity test (at standard dose every other day for 30 days). Data were mean ± SD (n = 3 independent samples).

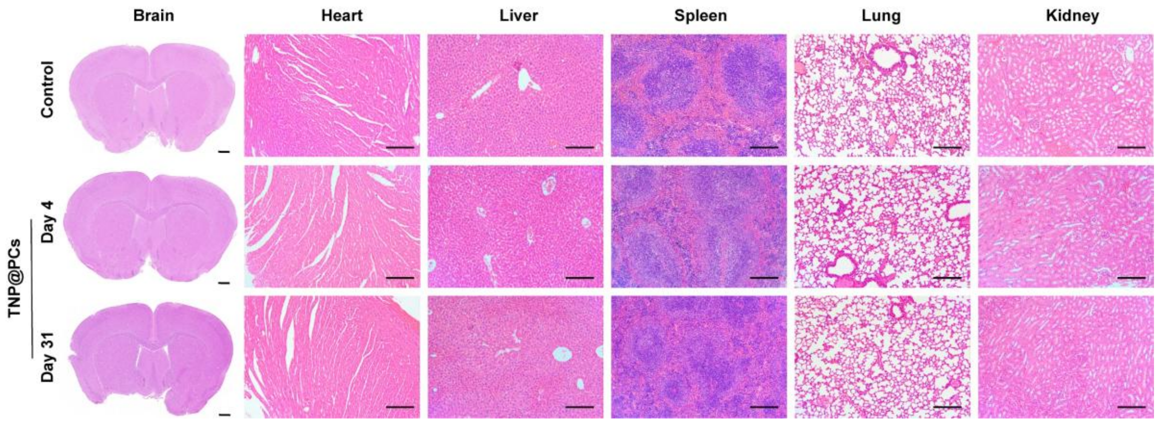

Fig. S19 H&E-stained major organs of healthy mice in acute and chronic toxicity tests (n = 3 independent samples). Scale bars: 500 μm.

Other supplementary datasets include the following:

Data S1. Proteins and relative abundances identified by LC-MS/MS.

Data S2. Gene expression levels of bulk RNA-seq data.

Data S3. DEGs from the bulk RNA-seq dataset.

Data S4. GO enrichment analysis based on GSEA.

Data S5. KEGG enrichment analysis based on GSEA.

Data S6. Gene expression data in transcripts per million (TPM) values.
